# Polarization of Vδ2 T cells to a Th2-like phenotype promotes plasmablast differentiation and possesses pro-fibrotic properties in IgG4-related disease

**DOI:** 10.3389/fimmu.2025.1550405

**Published:** 2025-03-27

**Authors:** Jieqiong Li, Mu Wang, Jiaxin Zhou, Yunyun Fei, Mengtao Li, Yan Zhao, Xiaofeng Zeng, Linyi Peng, Wen Zhang

**Affiliations:** ^1^ Department of Rheumatology and Clinical Immunology, Peking Union Medical College Hospital, Chinese Academy of Medical Sciences & Peking Union Medical College, National Clinical Research Center for Dermatologic and Immunologic Diseases, State Key Laboratory of Complex Severe and Rare Diseases, The Ministry of Education Key Laboratory, Beijing, China; ^2^ Department of Stomatology, Peking Union Medical College Hospital, Chinese Academy of Medical Sciences & Peking Union Medical College, Beijing, China

**Keywords:** IgG4-related disease, Vδ2 T cells, Th2-like phenotype, plasmablasts, IL-21–STAT3

## Abstract

**Objectives:**

To explore the phenotype and role of gamma delta (γδ) T cells in the pathogenesis of IgG4-related disease (IgG4-RD).

**Methods:**

Flow cytometry and quantitative RT-PCR were employed to analyze γδ T cell subsets, chemokine receptor expression, cytokine production, pro-fibrotic gene expression, and transcription factor profiles. Immunofluorescence assessed Vδ2 T cell infiltration in affected tissues. Chemotaxis assays and co-culture experiments investigated Vδ2 T cell migration and their influence on B cell differentiation. The impact of IL-21 stimulation and JAK/STAT3 inhibitors on γδ T cell was also evaluated.

**Results:**

Patients with IgG4-RD exhibited decreased peripheral Vδ2 T cells displaying a Th2-like phenotype characterized by elevated Th2 cytokine production and activated IL-21—STAT3—Blimp-1—GATA3 pathway. Vδ2 T cells accumulated in affected tissues through CCR7 upregulation, and co-localizing with B cells. Both Vδ2 T cells and culture supernatants from IgG4-RD patients promoted B cell differentiation. IL-21 stimulation augmented pSTAT3, Blimp-1, and GATA3 expression in Vδ2 T cells, while JAK and STAT3 inhibitors attenuated these effects. IgG4-RD patients exhibited increased TGF-β and pro-fibrotic gene expression in γδ T cells.

**Conclusion:**

Within the IL-21-rich microenvironment of IgG4-RD, peripheral Vδ2 T cells acquire a Th2-like phenotype via the IL-21—STAT3—Blimp-1—GATA3 pathway. Targeting JAK/STAT3 inhibitors holds therapeutic potential for IgG4-RD.

## Introduction

IgG4-related disease (IgG4-RD) is a systemic autoimmune disorder characterized by a fibrous-inflammatory process that can affect nearly any organ (1). Its pathogenesis remains poorly understood, and effective treatments for disease relapse are still limited. CD4+ cytotoxic T lymphocytes (CD4+CTLs) and B cells, including IgG4-expressing plasmablasts (PBs), represent the primary inflammatory cell populations implicated in causing organ damage and tissue fibrosis (2). IgG4-RD shares several pathogenic mechanisms with allergic diseases, such as T helper 2 (Th2)-dominated immune responses, hypersecretion of IgG4 and IgE, and the presence of blood and tissue eosinophilia (3). Although the adaptive immune system has long been considered central to IgG4-RD development, recent evidence has revealed the significance of the innate immune system. For instance, CD163+ M2 macrophages have been shown to promote fibrosis in IgG4-RD (4).

Gamma delta (γδ) T cells, an unconventional population of T lymphocytes crucial for host defense, immune surveillance, and immune system homeostasis (5), have recently emerged as key players in inflammatory and fibrotic diseases (6, 7). Based on their T cell receptor (TCR) δ-chain usage, γδ T cells are primarily divided into two subsets: Vδ1 and Vδ2. The Vδ2 T cells are the predominant γδ T cell subset in the peripheral blood of healthy adults, expanding after birth in response to host- or microbe-derived prenyl pyrophosphates, also known as phosphoantigens (pAgs) (8). Adding further complexity, Vδ2 T cells are functionally heterogeneous, resembling conventional T lymphocytes in their cytokine production and transcription factor expression. As such, Vδ2 T cells can be divided into functional subsets, including Th1, Th2, Th17, Th9, T follicular helper (Tfh), and regulatory T cells (Treg) (9).

γδ T cells are known to support B cells in antibody production and the maintenance of germinal centers (10). It has been reported that interleukin-21 (IL-21) enhances the potential of human γδ T cells to provide B-cell help by promoting the expression of markers associated with Tfh cells (11). However, whether this is also the case in IgG4-RD remains unclear, especially given that γδ T cells exhibit functional and phenotypic plasticity under different contexts. Several IL-21-regulated genes, including B lymphocyte-induced maturation protein-1 (Blimp-1), CXCR5, and Bcl-6, are critical in the immune response (12). Notably, in the context of IgG4-RD, IL-21—which serves as both an important B cell-helper cytokine and an autocrine factor—is predominantly produced by Tfh cells. IL-21 levels are elevated in the peripheral blood and tissues of patients with IgG4-RD, correlating with serum IgG4 levels (13). This observation suggests a potential role for γδ T cells in the pathogenesis of IgG4-RD, particularly in driving B cell differentiation.

Cell-cell communication through soluble and membrane-bound factors is critical in governing diverse cellular processes (14, 15). If γδ T cells indeed contribute to B cell help in IgG4-RD, as hypothesized, it will be important to elucidate the functional mechanisms underlying γδ T cell–B cell crosstalk. Interactions via CD40-CD40L and inducible co-stimulatory molecule (ICOS)-ICOSL are well-documented pathways involved in T-B communication, particularly in B cell activation and differentiation through direct cell-cell contact (16). Type 2 immune responses, classically characterized by a Th2 cytokine profile that includes IL-4, IL-5, and IL-13 (17), play a vital role in coordinating humoral immunity (18). However, the specific mechanisms of γδ T cell–B cell interaction in IgG4-RD remain unknown.

To date, no studies have explored the role of γδ T cells in IgG4-RD. In this study, we demonstrate the potential involvement of Vδ2 T cells in the pathogenesis of IgG4-RD. Our findings reveal that Vδ2 T cells infiltrate affected tissues, exhibit a Th2-like phenotype, and contribute to B cell help that promotes PB differentiation. Mechanistically, our *in vitro* experiments indicate that these effects are mediated via an IL-21–STAT3–Blimp-1 circuit, which leads to the upregulation of GATA3 and a Th2 phenotype. Furthermore, we show that JAK and STAT3 inhibitors can interrupt this process. Taken together, this study uncovers a novel role for Vδ2 T cells in IgG4-RD, providing insights into its pathogenesis and identifying potential therapeutic targets for this disease.

## Methods

### Patients

Peripheral blood mononuclear cells (PBMCs) were obtained from patients with IgG4-related disease (IgG4-RD) who met the 2019 classification criteria established by the American College of Rheumatology/European League Against Rheumatism for IgG4-RD (19). Age- and sex-matched healthy controls (HCs) were included for comparison. The study enrolled 40 patients with IgG4-RD and 16 HCs. Detailed clinical characteristics of the IgG4-RD patients are provided in **Supplementary Table S1**. Submandibular gland biopsies were collected from 3 patients with active IgG4-RD and 3 controls with chronic sialadenitis. The study was approved by the Ethics Committee of Peking Union Medical College Hospital (approval number: K3738), and informed consent was obtained from all participants.

### Pre-expansion of Vδ2 T cells

PBMCs were isolated from heparinized blood using density gradient centrifugation with human lymphocyte separation solution. γδ T cells were purified from PBMCs using the Human anti-TCR γ/δ MicroBead Kit (Miltenyi Biotec, Bergisch Gladbach, Germany), achieving a purity of >90% (**Supplementary Figure S1A**).

Purified γδ T cells were resuspended in RPMI-1640 medium supplemented with 10% fetal bovine serum (FBS), 100 IU/ml penicillin, 100 µg/ml streptomycin, and 100 mM sodium pyruvate (Institute of Basic Medical Sciences, Chinese Academy of Medical Sciences, Beijing, China). The cells were seeded in 96-well round-bottom plates (Corning-Costar, Corning, NY, USA) at a density of 5×10^4^ to 1×10^5^ cells per well. To expand the Vδ2+ subset, γδ T cells were stimulated with isopentenyl pyrophosphate (IPP) (20 µg/ml, Sigma Chemical Co., St. Louis, MO, USA) and IL-2 (PeproTech, Cranbury, NJ, USA). The purity of Vδ2 T cells after expansion exceeded 90% (**Supplementary Figure S1B**). Cells were incubated at 37°C with 5% CO_2_, and the medium was replenished every 3 days by adding fresh IL-2-supplemented medium.

### Induction of Th2-like phenotype

To induce a Th2-like phenotype in γδ T cells, 96-well round-bottom plates were pre-coated with anti-human CD3 (2.0 µg/ml, BD Biosciences, Franklin Lakes, NJ, USA) for 4 hours at 37°C. γδ T cells were isolated, counted, and seeded at a density of 5×10^4^ to 1×10^5^ cells per well in 200 µl of culture medium. Th2 polarization was induced by stimulating cells with anti-CD3 (2.0 µg/ml), anti-CD28 (4 µg/ml, BD Biosciences), IPP (20 µg/ml, Sigma Chemical Co.), IL-4 (20 ng/ml, PeproTech), and IL-2 (10 ng/ml, PeproTech). The culture medium was replenished on day 3, and cells were harvested on day 6. Th2-associated transcription factor GATA3 expression was assessed by flow cytometry.

### Co-culture of γδ T cells and B cells

γδ T cells from IgG4-RD patients and HCs were isolated by magnetic bead positive selection (Miltenyi Biotec). B cells from HCs were isolated using magnetic bead negative selection (B Cell Isolation Kit II, Miltenyi Biotec), achieving >90% purity. Co-culture experiments were performed in 96-well round-bottom plates pre-coated with anti-human CD3 (1.0 µg/ml, BD Biosciences). γδ T cells and B cells were co-cultured at a 1:1 ratio (5×10^4^ cells/well) in the presence of anti-CD28 (4 µg/ml, BD Biosciences), IPP (20 µg/ml, Sigma Chemical Co.), IL-2 (10 ng/ml, PeproTech), CD40L (100 ng/ml, PeproTech), and CpG (200 ng/ml, InvivoGen, Toulouse, France). Half of the culture medium was replaced on day 3, and supernatants were collected and stored at -80°C. After 6 days, culture supernatants were analyzed for IgG, IgG4, and IgE levels by ELISA (R&D Systems, Minneapolis, MN, USA). B cell subsets were analyzed by flow cytometry.

### Culture of B cells with Vδ2 T cell supernatant

Vδ2 T cells from IgG4-RD patients and HCs were expanded following the pre-expansion protocol. On day 6, culture supernatants from Vδ2 T cells (Vδ2 T-SN) were collected and used to stimulate HC-derived B cells. B cell culture supernatants were collected on day 6 for immunoglobulin quantification by ELISA, while B cell subsets were analyzed by flow cytometry.

### Flow cytometry (FACS) analysis

Flow cytometry was used to analyze the percentages of γδ T cells, Vδ1 T cells, Vδ2 T cells, B cells, plasmablasts, chemokine receptors, intracellular cytokines, and transcription factors. Fluorescently labeled antibodies and isotype controls were used (**Supplementary Table S2**). Intracellular staining and phospho-flow analysis were performed using fixation and permeabilization buffers (BD Biosciences), and nuclear transcription factor staining was conducted with the Foxp3/Transcription Factor Staining Buffer Set (eBioscience). Data were acquired using a FACSAria II system (BD Biosciences) and analyzed with FlowJo software (version X, FlowJo, Ashland, OR, USA).

### Real-time quantitative PCR (qPCR)

Total RNA was extracted from γδ T cells using the RNA-Quick Purification Kit (ES Science, Shanghai, China). Reverse transcription and qPCR were performed using the PrimeScript™ RT Master Mix and TB Green™ Premix Ex Taq™ II kits (Takara Bio) according to the manufacturer’s instructions. Relative mRNA expression was calculated using the 2^-ΔΔCt method with housekeeping genes as internal controls. Gene-specific primers were designed based on GenBank sequences and published studies (**Supplementary Table S3**).

### Transwell migration assays

Transwell assays were conducted to evaluate the migratory capacity of γδ T cells in response to CCL21. γδ T cells from IgG4-RD patients and HCs were seeded in the upper chambers of 24-well Transwell plates (Corning-Costar). CCL21 (PrimeGene Bio-Tech, Shanghai, China) was added to the lower chambers at concentrations of 50 ng/ml and 500 ng/ml. Cells were allowed to migrate for 4 hours at 37°C in a 5% CO_2_ incubator. Migrated cells were quantified using flow cytometry and 123count eBeads™ (Invitrogen).

### Stimulation and inhibition experiments

To investigate the role of IL-21 signaling in Vδ2 T cell differentiation, γδ T cells were stimulated with IL-21 (100 ng/ml, PeproTech) or treated with JAK/STAT3 pathway inhibitors, including Baricitinib (300 nM), Tofacitinib (10 nM and 100 nM), and STAT3 inhibitor S3I-201 (NSC 74859) (Selleck). Protein phosphorylation and transcription factor expression (e.g., pSTAT3, GATA3, and Blimp-1) were analyzed by flow cytometry.

### Immunofluorescence

Immunofluorescence staining was performed using primary antibodies against CD20, TCR Vδ2, CCR7, and CCL21 (Abcam, Affinity). DAPI was used for nuclear counterstaining.

### Statistical analysis

Statistical analyses were performed using Prism software. To assess the normality of data distribution, the Shapiro-Wilk test were applied. Data with normal distribution and homogeneity of variance were analyzed using independent t-tests (two groups) or one-way ANOVA (multiple groups) with LSD or SNK for *post-hoc* comparisons. Non-normally distributed data were analyzed using the Mann-Whitney U test (two groups) or the Kruskal-Wallis test (multiple groups). In the figures, box plots represent the median [min to max range], while scatter plots with error bars represent the mean ± SD. We used MFI (Median Fluorescence Intensity or Mean Fluorescence Intensity) to describe the fluorescence intensity of cells or particles analyzed by flow cytometry. For gates where the fluorescence intensities were normally distributed, we used Mean Fluorescence Intensity; otherwise, Median Fluorescence Intensity was applied. Correlations were assessed using Pearson or Spearman correlation coefficients. Categorical variables were compared using chi-square tests. A p-value <0.05 was considered statistically significant.

## Results

### Circulating Vδ2 T cells are decreased in IgG4-RD patients and correlate with clinical parameters

**Figure 1:**
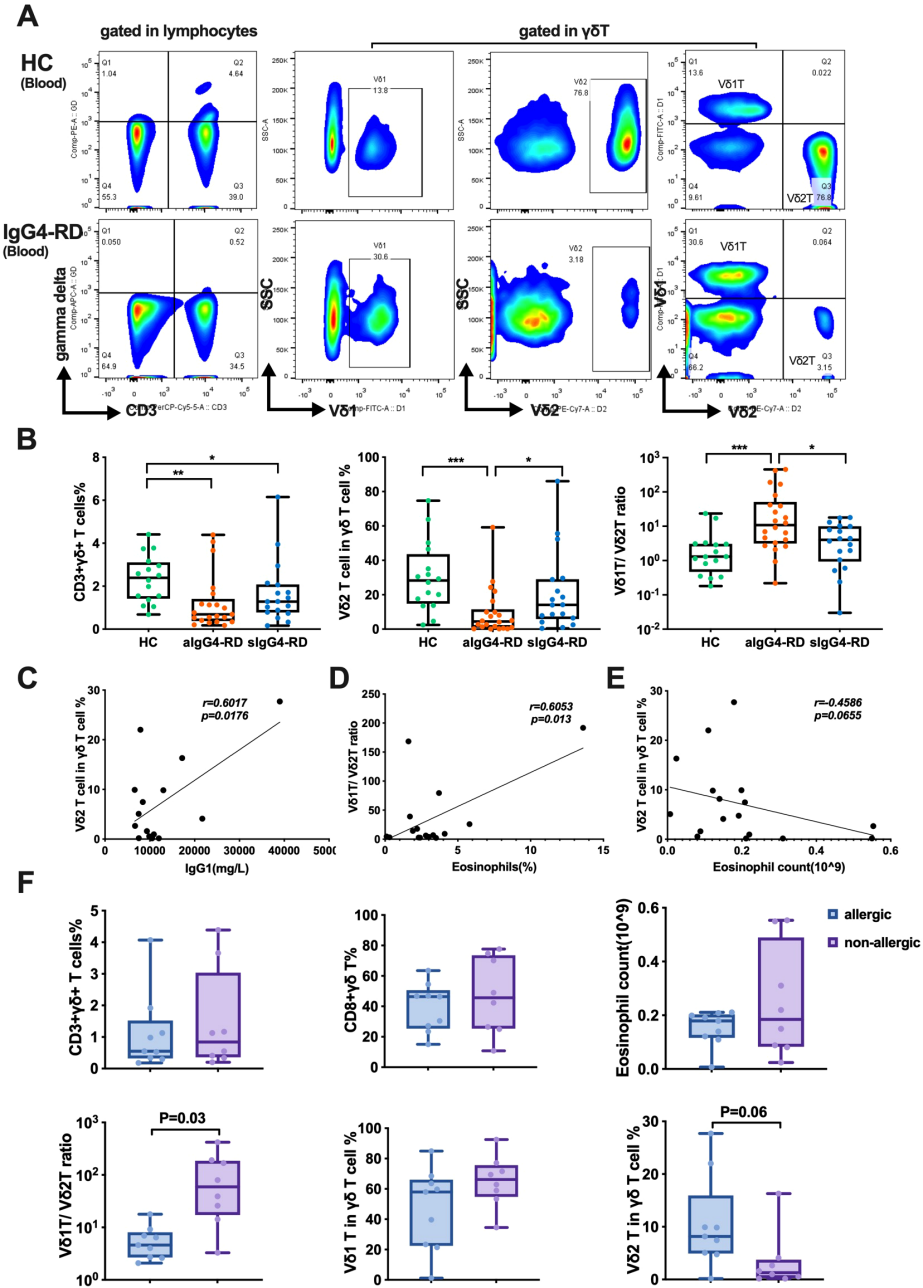
The proportions of γδ T cells and their subsets in peripheral blood and correlations with clinical parameters. **(A)** The gating strategy to identify γδ T cells. **(B)** Comparison of the proportions of γδ T cells and their subsets among patients with active IgG4-RD (aIgG4-RD), stable IgG4-RD (sIgG4-RD), and healthy controls (HC). Box plots show the distribution of CD3+γδ+ T cells, Vδ2 T cells, and the Vδ1/Vδ2 T cell ratio across the three groups. **(C)** Positive correlation between the proportion of Vδ2 T cells and serum IgG1 level. **(D)** Positive correlation between the Vδ1/Vδ2 T cell ratio and the percentage of eosinophils in peripheral blood. **(E)** Correlation analysis between Vδ2 T cells and the absolute number of peripheral blood eosinophils. **(F)** Comparison of the proportion of γδ T cells and their subsets, and the absolute number of eosinophils in peripheral blood between IgG4-RD patients with allergic diseases (allergic) and those without allergic diseases (non-allergic). Box plots illustrate differences in CD3+γδ+ T cells, CD8+γδ+ T cells, eosinophil counts, Vδ1/Vδ2 T cell ratio, Vδ1 T cells, and Vδ2 T cells between allergic and non-allergic groups. *P < 0.05, **P < 0.01, ***P < 0.001.

To investigate the potential contribution of γδ T cells to the pathogenesis of IgG4-RD, we first analyzed the total γδ T cells and their subpopulations in the peripheral blood of patients with active IgG4-RD (aIgG4-RD, n=22), stable IgG4-RD (sIgG4-RD, n=18), and age- and sex-matched healthy controls (HCs, n=16). The gating strategy used to identify γδ T cells in the peripheral blood is shown in **Figure 1A**. The frequency of total γδ T cells (CD3+γδ+) was significantly lower in aIgG4-RD compared with HCs (1.218% ± 0.28% vs. 2.34% ± 0.27%, p<0.01). In particular, the percentage of Vδ2 T cells within the total γδ T cell population was approximately three times lower in aIgG4-RD compared with HCs (9.52% ± 2.95% vs. 30.15% ± 5.05%, p<0.001) (**Figure 1B**). Consequently, the Vδ1 T/Vδ2 T cell ratio was markedly increased in aIgG4-RD patients compared with HCs (**Figure 1B**). However, there were no significant differences in the frequencies of Vδ1 T cells or CD8+γδ T cells between aIgG4-RD and HCs (**Supplementary Figure S2A**). Interestingly, Vδ2 T cell frequencies were restored in sIgG4-RD patients following treatment (21.03% ± 5.36%) (**Figure 1B**).

To evaluate the clinical relevance of these findings, we analyzed correlations between γδ T cell frequencies and various clinical and serological parameters. The proportion of Vδ2 T cells positively correlated with serum IgG1 levels (r=0.6017, p=0.0176) (**Figure 1C**), while the percentage of circulating Vδ1 T cells negatively correlated with serum IgG3 levels (r=-0.6372, p=0.0106) (**Supplementary Figure S2B**). Additionally, the Vδ1 T/Vδ2 T cell ratio showed a positive correlation with peripheral blood eosinophil percentages (r=0.6053, p=0.013) (**Figure 1D**). The eosinophil count was inversely correlated with the frequency of Vδ2 T cells (r=-0.4586, p=0.0655) (**Figure 1E**). No significant correlations were observed between Vδ2 T cell frequencies and the number of involved organs, serum IgG, IgG4, total IgE, CRP, ESR, or the IgG4-RD responder index (RI) (data not shown).

When comparing patients with or without allergic disease, the allergic group exhibited a significantly decreased Vδ1 T/Vδ2 T cell ratio (6.27 ± 1.65 vs. 117.9 ± 49.99, p<0.05). However, no differences were observed in the percentages of total γδ T cells, Vδ1 T cells, CD8+γδ T cells, or eosinophil counts between the two groups (**Figure 1F**).

Collectively, these results indicate that patients with aIgG4-RD are characterized by a significant reduction in circulating Vδ2 T cells and an increased Vδ1 T/Vδ2 T cell ratio, which correlate with certain clinical and serological parameters.

### Type-2 cytokines are increased in Vδ2 T cells from IgG4-RD patients

**Figure 2:**
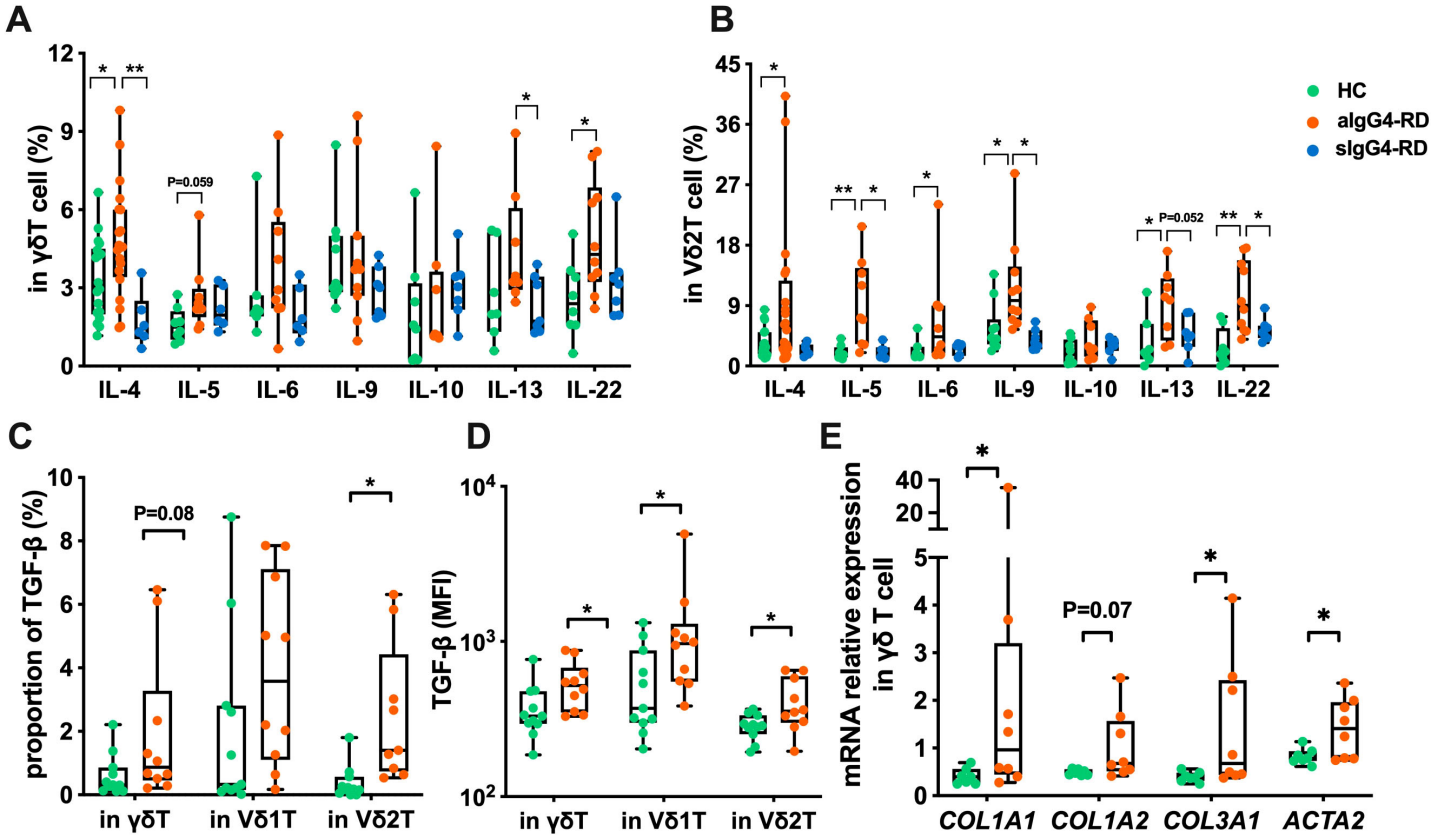
Compare the expression of cytokines and activation of pro-fibrotic pathways in γδ T cells of IgG4-RD patients and healthy controls (HC). **(A)** Comparison of expression levels of Th2-type cytokines (IL-4, IL-5, IL-6, IL-9, IL-10, IL-13, IL-22) in γδ T cells between HC, active IgG4-RD (aIgG4-RD), and stable IgG4-RD (sIgG4-RD) patients. **(B)** Comparison of expression levels of Th2-type cytokines in Vδ2 T cells among HC, aIgG4-RD, and sIgG4-RD patients. **(C)** Comparison of the proportion of TGF-β expressing cells in γδ T cells and their subsets (Vδ1 T cells and Vδ2 T cells) between HC and IgG4-RD patients. **(D)** Comparison of MFI values of TGF-β expression in peripheral blood γδ T cells and their subsets between HC and IgG4-RD patients. **(E)** Comparison of mRNA expression levels of pro-fibrotic related molecules (*COL1A1*, *COL1A2*, *COL3A1*, *ACTA2*) in γδ T cells between HC and IgG4-RD patients. *P < 0.05, **P < 0.01, ***P < 0.001.

To investigate the polarization of γδ T cells, particularly Vδ2 T cells, in IgG4-RD patients, we measured the expression of type-1, type-2, and type-17 cytokines. While no significant differences were observed in the expression or MFI of IFN-γ, TNF-α, or IL-17A between aIgG4-RD patients and HCs (**Supplementary Figure S3**), total γδ T cells from aIgG4-RD patients produced higher levels of IL-4 and IL-22, with a trend toward increased IL-5 compared to HCs. Following treatment, IL-4 and IL-13 production was significantly reduced in γδ T cells from sIgG4-RD patients compared to aIgG4-RD patients (**Figure 2A**).

Focusing on Vδ2 T cells, we found increased expression of Th2 cytokines, including IL-4, IL-5, IL-6, IL-9, and IL-13, as well as IL-22 (a member of the IL-10 family), in aIgG4-RD patients compared to HCs. However, IL-10 expression was comparable across all groups (**Figure 2B**). After treatment, Vδ2 T cells from sIgG4-RD patients exhibited reduced expression of IL-5, IL-9, IL-13, and IL-22 compared to aIgG4-RD patients (**Figure 2B**). These findings suggest that type-2 cytokines are elevated in γδ T cells, particularly Vδ2 T cells, in IgG4-RD and are significantly reduced after treatment.

We also assessed the expression of transforming growth factor-beta (TGF-β), a key Th2 cytokine implicated in fibrosis due to its ability to activate and induce fibroblast proliferation (20, 21). Both the proportion and MFI of TGF-β expression in Vδ2 T cells were significantly higher in IgG4-RD patients compared to HCs (**Figures 2C, D**).

To further confirm the activation of pro-fibrotic pathways, we performed quantitative PCR to measure the expression of α-smooth muscle actin (*ACTA2*), *COL1A1*, *COL1A2*, and *COL3A1* collagen genes, which have been reported to be elevated in IgG4-RD (22). γδ T cells from IgG4-RD patients showed significantly increased expression of *COL1A1*, *COL3A1*, and *ACTA*2, but not *COL1A2*, suggesting a potential role for γδ T cells in the pro-fibrotic processes associated with IgG4-RD pathogenesis (**Figure 2E**).

Overall, these results demonstrate that Vδ2 T cells in IgG4-RD exhibit increased production of type-2 cytokines, supporting their polarization toward a Th2-like phenotype, which may contribute to the aberrant humoral immune responses observed in IgG4-RD.

### Circulating Vδ2 T cells display an intrinsic Th2-like phenotype in IgG4-RD

**Figure 3:**
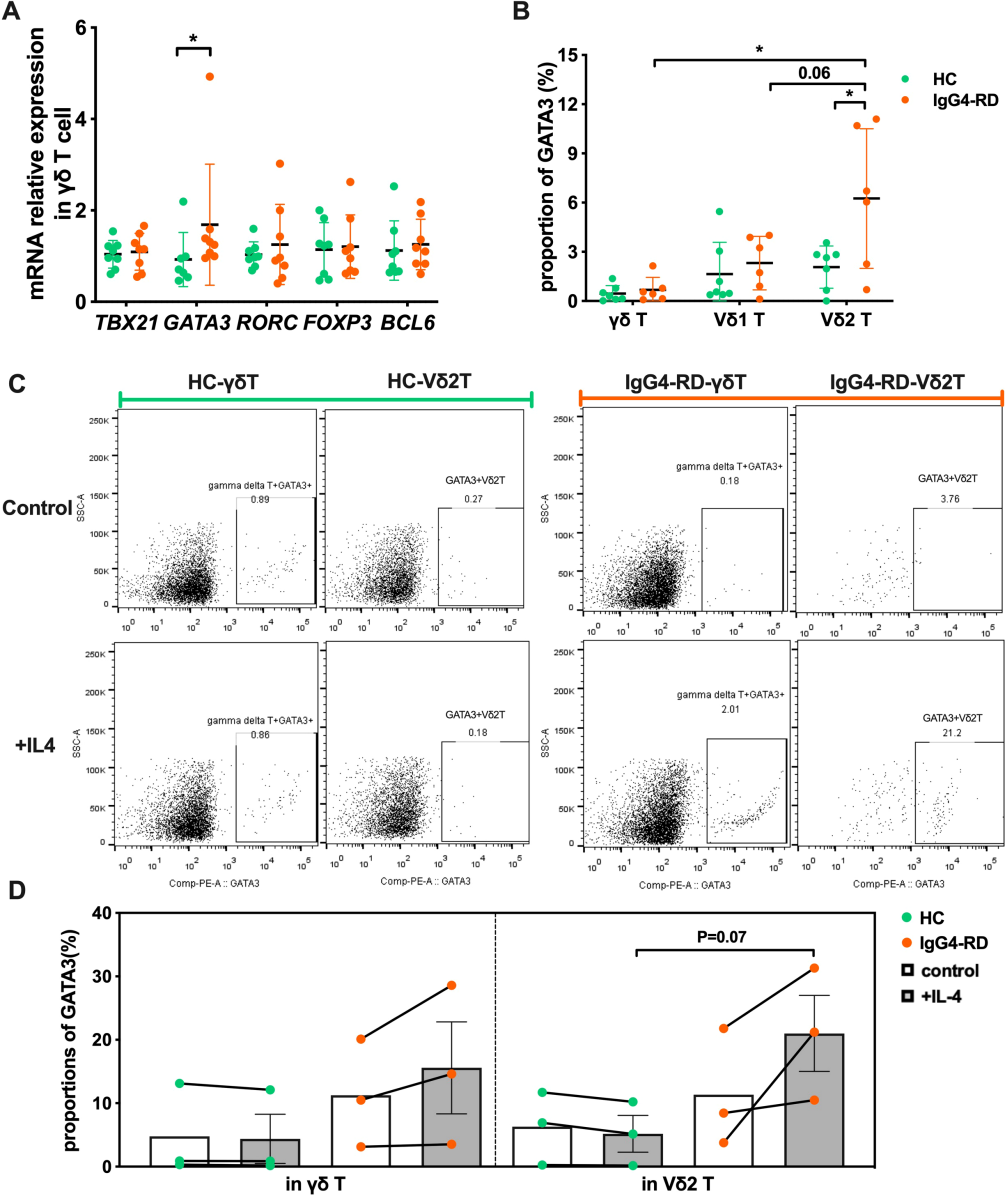
γδ T cells in patients with IgG4-RD have an intrinsic Th2-like phenotype. **(A)** Comparison of mRNA expression levels of transcription factors *TBX21*, *GATA3*, *RORC*, *FOXP3*, and *BCL6* in γδ T cells between healthy controls (HC) and IgG4-RD patients. **(B)** Comparison of the protein expression ratio of GATA3 in γδ T cells and their subsets (Vδ1 T cells and Vδ2 T cells) between HC and IgG4-RD patients. **(C)** Flow cytometry analysis showing the IL-4-induced Th2-like phenotype (GATA3 expression) in γδ T cells of HC and IgG4-RD patients. **(D)** Comparison of IL-4-induced GATA3 expression in γδ T cells between HC and IgG4-RD patients. “Control” refers to the control group without IL-4 treatment, and “+IL-4” refers to the experimental group treated with IL-4. *P < 0.05, **P < 0.01, ***P < 0.001.

T-bet, GATA3, RORγt, BCL-6, and Foxp3 are major transcription regulators of Th1, Th2, Th17, Tfh, and Treg cells, respectively (23, 24). To further understand the intrinsic nature of Vδ2 T cell subset in IgG4-RD, we measured the mRNA expression levels of *TBX21* (Th1), *GATA3* (Th2), *RORC* (Th17), *BCL-6* (Tfh) and *FOXP3* (Treg) in peripheral blood γδ T cells. As expected, Th2-associated *GATA3* expression was increased (P<0.05) in IgG4-RD patients, while expressions of *TBX21*, *RORC*, *BCL-6* and *FOXP3* were comparable between HCs and IgG4-RD patients (**Figure 3A**). We then confirmed these results at the protein level (**Figure 3B**). A significant increase GATA3 was observed in Vδ2 T cells from patients with IgG4-RD, while no difference of expression was observed for GATA3 in γδ T cells nor Vδ1 T cells. These observations suggest that the Vδ2 T cells in IgG4-RD presented a salient Th2-like phenotype.

IL-4 is a potent inducer that directs differentiation of naive T cells into Th2 effector cells (25). We therefore investigated the Th2 phenotype polarity of Vδ2 T cells to IL-4 stimulation. Vδ2 T cells from IgG4-RD responded positively, and we observed that GATA3 expression was approximately two times higher in IL-4-stimulated Vδ2 T cells compared with non-stimulation (11.34% ± 5.40% vs 21.00% ± 6.01%) in the patients with IgG4-RD, together with a slightly increase in IL-4-stimulated total γδ T cells (11.24% ± 4.91% vs 15.58% ± 7.25%) (**Figure 3C**). However, the stimulation didn’t effective for total γδ T cells and Vδ2 T cells in HCs (4.77% ± 4.17% vs 4.38% ± 3.87%, 6.29% ± 3.31% vs 5.17% ± 2.89%) (**Figure 3C**). GATA3^+^ Vδ2 T cells frequency was also increased in IgG4-RD compared with HCs after IL-4 stimulation (21.00% ± 6.01% vs 5.17% ± 2.89%, P=0.07) (**Figure 3D**).

Collectively, our data show that Vδ2 T cells in IgG4-RD favors the intrinsic polarity of Th2-like phenotype characterized by high GATA3 expression, which simultaneously results in their enhanced type-2 cytokines production capacity.

### CCR7 facilitates the chemotaxis of circulating Vδ2 T cells to involved tissues and their co-localization with B cells via the CCR7/CCL21 axis

Chemokine receptors and their ligands play a crucial role in the migration and positioning of immune cells (26), particularly in the infiltration of immune cells into affected tissues in autoimmune diseases (27). Given the observed reduction of circulating Vδ2 T cells in IgG4-RD, we hypothesized that these cells may migrate into disease-involved tissues.

**Figure 4:**
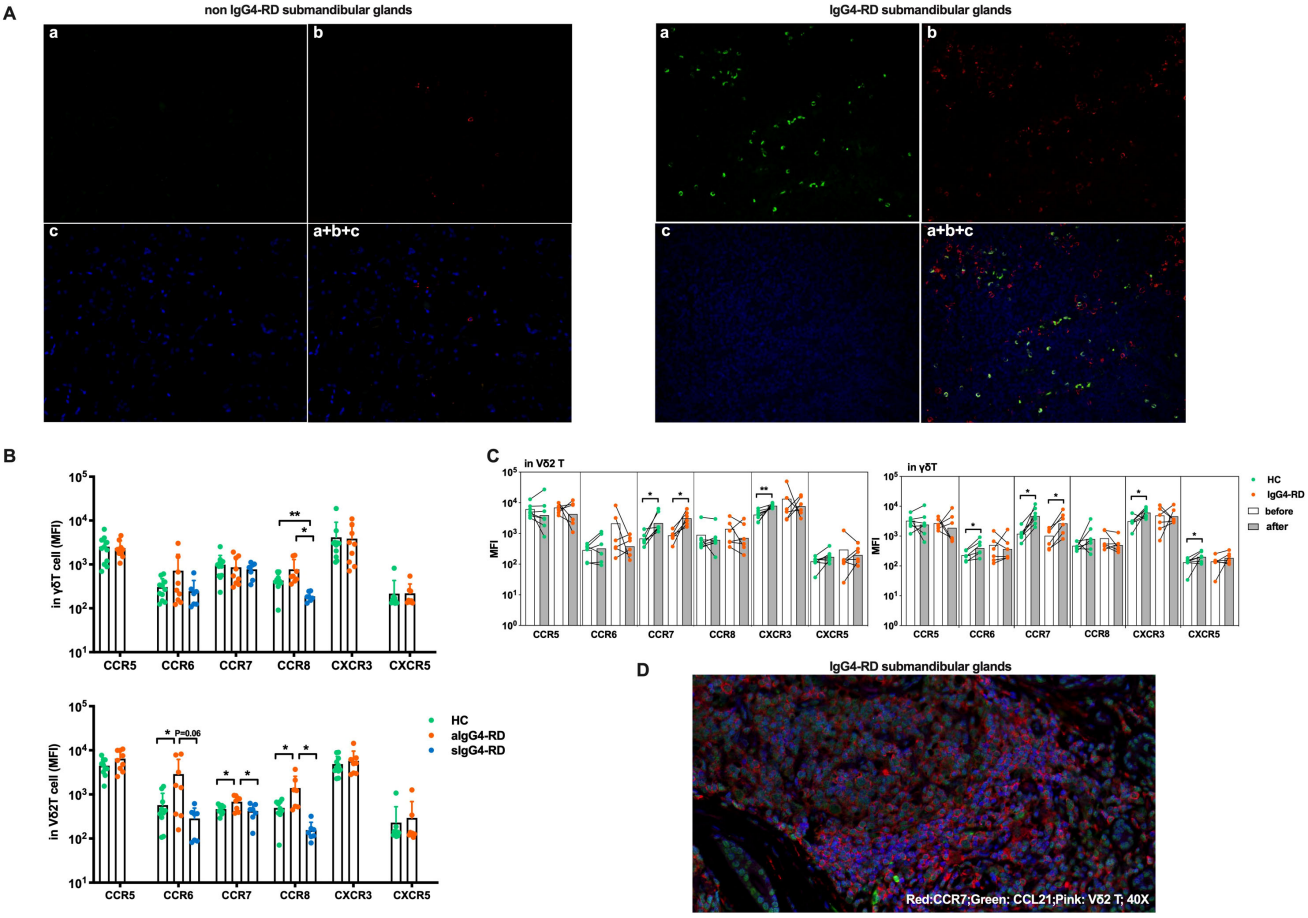
Circulating Vδ2 T cells migrate to involved tissues through CCR7. **(A)** Immunofluorescence results showing that submandibular glands affected by IgG4-RD have more Vδ2 T cells infiltrating than those affected by chronic sialadenitis, and these Vδ2 T cells colocalize with B cells. a, Vδ2 T (green); b, CD20 (red); c, DAPI counterstaining for cell nuclei (blue); a + b + c, Merged image of Vδ2 T, CD20, and DAPI; Magnification: 20×. **(B)** MFI levels of chemokine receptors (CCR5, CCR6, CCR7, CCR8, CXCR3, and CXCR5) on the surface of γδ T cells and Vδ2 T cells in active IgG4-RD (aIgG4-RD) patients, healthy controls (HC), and stable IgG4-RD (sIgG4-RD) patients. The expression levels of CCR6, CCR7, and CCR8 are also shown after treatment. **(C)** Comparison of changes in the MFI levels of chemokine receptors on the surface of γδ T cells and Vδ2 T cells before and after *in vitro* IPP + IL-2 stimulation. **(D)** Immunofluorescence results demonstrating that Vδ2 T cells, CCR7, and CCL21 colocalize in the submandibular gland tissue affected by IgG4-RD. Blue: DAPI (nucleus). *P < 0.05, **P < 0.01.

To assess this, we performed multicolor immunofluorescence staining on submandibular gland tissues from IgG4-RD patients. As shown in **Figure 4A**, significantly increased infiltration of Vδ2 T cells was observed in IgG4-RD submandibular glands compared with controls. Additionally, Vδ2 T cells and CD20^+^ B cells were found in close proximity within IgG4-RD-affected tissues (**Figure 4A**). Similar observations were also noted in the lacrimal glands (**Supplementary Figure S4**). These findings suggesting potential interactions between these cell types at the disease site.

To explore whether the reduction in circulating Vδ2 T cell numbers was due to enhanced tissue chemotaxis, we conducted a chemokine receptor expression profile screening. We found significantly higher expression levels of CCR6, CCR7, and CCR8 on Vδ2 T cells from aIgG4-RD patients compared with HCs, and these levels were restored after treatment in sIgG4-RD patients (p<0.05, **Figure 4B**, **Table 1**). To evaluate the persistence of chemokine receptor expression, Vδ2 T cells were cultured in the presence of IPP and IL-2 for three days. Interestingly, only CCR7 expression was significantly upregulated in γδ T cells and Vδ2 T cells from IgG4-RD patients (p<0.05, **Figure 4C**).

**Table 1:** Expression levels of chemokine receptors on γδ T cells and Vδ2 T cells.

MFI	HC	aIgG4-RD	sIgG4-RD	P value		HC	aIgG4-RD	sIgG4-RD	P value	
	γδ T			t-test	ANOVA	Vδ2 T			t-test	ANOVA
**CCR5**	2537 ± 1690	2355 ± 1086	NA	0.7831	NA	4467 ± 1835	6505 ± 3132	NA	0.0976	NA
**CCR6**	305.1 ± 166.6	719.1 ± 973.1	247 ± 185.5	0.1801	0.1996	571.7 ± 487.6	2894 ± 3322	285 ± 204.8	**0.0431**	**0.0227**
**CCR7**	985.5 ± 614.3	859.2 ± 610.3	774.7 ± 299	0.6521	0.7207	467.3 ± 106.4	683.9 ± 235.1	421.9 ± 170.2	**0.0246**	**0.0175**
**CCR8**	431.3 ± 170.1	773.4 ± 515.7	187.9 ± 44.75	0.0651	**0.0052**	494.1 ± 217.5	1400 ± 1171	155.6 ± 78.54	**0.0379**	**0.0056**
**CXCR3**	4154 ± 5036	3866 ± 3562	NA	0.8868	NA	4878 ± 2367	5790 ± 3783	NA	0.5264	NA
**CXCR5**	216.1 ± 214.4	219.4 ± 140.7	NA	0.9708	NA	230.7 ± 296.7	293.6 ± 397.8	NA	0.7053	NA

The bold values indicate P < 0.05.

To examine the chemotactic capabilities of Vδ2 T cells mediated by the CCR7 in IgG4-RD, we performed an *in vitro* transwell migration assay. As expected, Vδ2 T cells from IgG4-RD patients demonstrated a significantly higher migration rate in response to the CCL21 chemokine gradient compared to HCs, while the migration rates of γδ T cells as a whole were similar between the two groups (**Supplementary Figure S5A, B**). Notably, the proportion of Vδ2 T cells in the lower chambers relative to the upper chambers was significantly higher in IgG4-RD patients (81.70%) compared to HCs (66.08%) (**Supplementary Figure S5C**). Furthermore, Vδ2 T cells constituted a higher percentage of cells in the lower chambers in IgG4-RD patients (82.9%) than in HCs (54.2%), while Vδ1 T cells were less frequent in the lower chambers in IgG4-RD patients (5.14%) compared to HCs (24.1%) (**Supplementary Figure S5D, E**). These findings suggest an enhanced migration of Vδ2 T cells toward CCL21 driven by CCR7 in IgG4-RD, consistent with the significant upregulation of CCR7 on these subsets.

Additionally, CCR7 was found to be highly expressed on Vδ2 T cells infiltrating IgG4-RD lesions, and these cells were co-localized with CCL21 in the affected tissues (**Figure 4D**). These findings align with the observation of increased CCR7 expression supporting the tissue migration of Vδ2 T cells in IgG4-RD.

In summary, our data indicate that peripheral Vδ2 T cells infiltrate IgG4-RD lesions at least in part via the CCR7/CCL21 axis and potentially engage in crosstalk with infiltrated B cells. These interactions may play a critical role in the pathogenesis of IgG4-RD.

### Vδ2 T cells from IgG4-RD patients promote B-cell differentiation and immunoglobulin production in IgG4-RD

**Figure 5:**
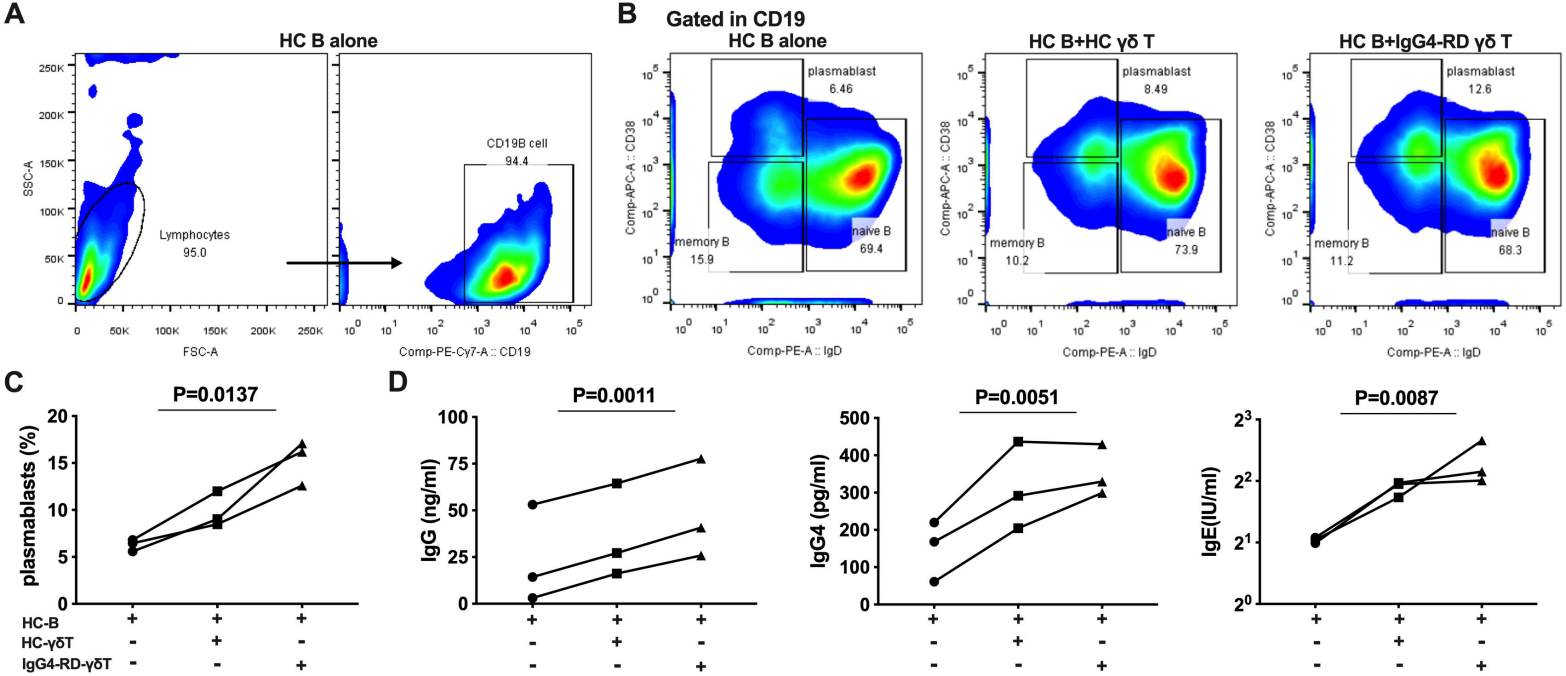
γδ T cells from IgG4-RD patients promote B cells to differentiate into plasmablasts and secrete immunoglobulins. **(A)** Flow cytometric analysis of the proportion of CD19+ B cells after 6 days of culture of healthy control (HC) B cells alone. **(B)** Flow cytometric analysis of the proportion of plasmablasts in three conditions: HC B cells alone, HC B cells co-cultured with HC γδ T cells, and HC B cells co-cultured with γδ T cells from IgG4-RD patients. **(C)** Comparison of the proportion of plasmablasts. Co-culture of HC B cells with γδ T cells from IgG4-RD patients promotes B cell differentiation into plasmablasts more than HC B cell culture alone or co-culture with HC γδ T cells. **(D)** ELISA detection of immunoglobulin levels (IgG, IgG4, and IgE) in the supernatant of γδ T cells and B cells co-cultures. The levels of immunoglobulins are higher in co-cultures involving γδ T cells from IgG4-RD patients compared to those involving HC γδ T cells or HC B cell culture alone.

To evaluate the B-cell helper properties of Vδ2 T cells in patients with IgG4-RD, we established co-culture experiments using B cells and Vδ2 T cells. Pre-expanded Vδ2 T cells from patients with active IgG4-RD (aIgG4-RD) or healthy controls (HCs) were cultured with magnetic bead–purified B lymphocytes from healthy donors for six days (**Figure 5A**). B cells cultured alone served as the control group.

We observed a significant increase in the proportion of CD19+IgD-CD38high plasmablasts in co-cultures of B lymphocytes with Vδ2 T cells compared to B cells cultured alone (15.3% ± 2.38% vs. 6.28% ± 0.63%). Moreover, Vδ2 T cells from IgG4-RD patients promoted B cell differentiation into CD19+IgD-CD38high plasmablasts more effectively than Vδ2 T cells from HCs (15.3% ± 2.38% vs. 9.84% ± 1.89%) (**Figures 5B, C**).

We further quantified the levels of immunoglobulin in the supernatants of the three culture conditions. Consistently, co-cultures of B cells with Vδ2 T cells from IgG4-RD patients showed significantly higher production of immunoglobulins compared to the co-cultures involving Vδ2 T cells from HCs and B cells cultured alone. This included increases in total IgG (ng/ml) (48.12 ± 15.42 vs. 35.93 ± 14.59 vs. 23.52 ± 15.15, P<0.01), IgG4 (pg/ml) (352.8 ± 39.45 vs. 310.80 ± 67.68 vs. 149.6 ± 46.69, P<0.01), and IgE (IU/ml) (4.93 ± 0.71 vs. 3.70 ± 0.19 vs. 2.06 ± 0.04, P<0.01) (**Figure 5D**).

In summary, these co-culture experiments demonstrated that Vδ2 T cells from IgG4-RD patients exhibit a higher capacity than those from HCs to promote plasmablast differentiation and enhance immunoglobulin production. These findings suggest a central role for Vδ2 T cells in augmenting B-cell activity and humoral responses in IgG4-RD.

### Vδ2 T cells from IgG4-RD patients provide B-cell help through type 2 immune response

To determine how Vδ2 T cells from IgG4-RD patients promote B cell differentiation, we investigated whether Vδ2 T cells interact with B lymphocytes via a Th2-like phenotype or adopt Tfh-like functions, as Tfh cells are known to play a key role in activating B cells and guiding their differentiation (28, 29). We assessed the expression of Tfh-associated markers in Vδ2 T cells, including transcriptional regulators (BCL6), surface markers (CXCR5, ICOS, and CD40L), and cytokine production profiles (IL-21 and IL-6). No significant differences were observed between patients with IgG4-RD and healthy controls (HCs) regarding IL-21 expression (**Supplementary Figure S6A**) or the expression of the B-cell costimulatory molecules CD40L and ICOS (**Supplementary Figure S6B, C**). These results, along with data showing no upregulation of CXCR5 and BCL6 in Vδ2 T cells from IgG4-RD patients (**Figures 3A**, **4A**), ruled out the possibility that the Tfh phenotype in Vδ2 T cells contributes to B-cell help in IgG4-RD.

**Figure 6:**
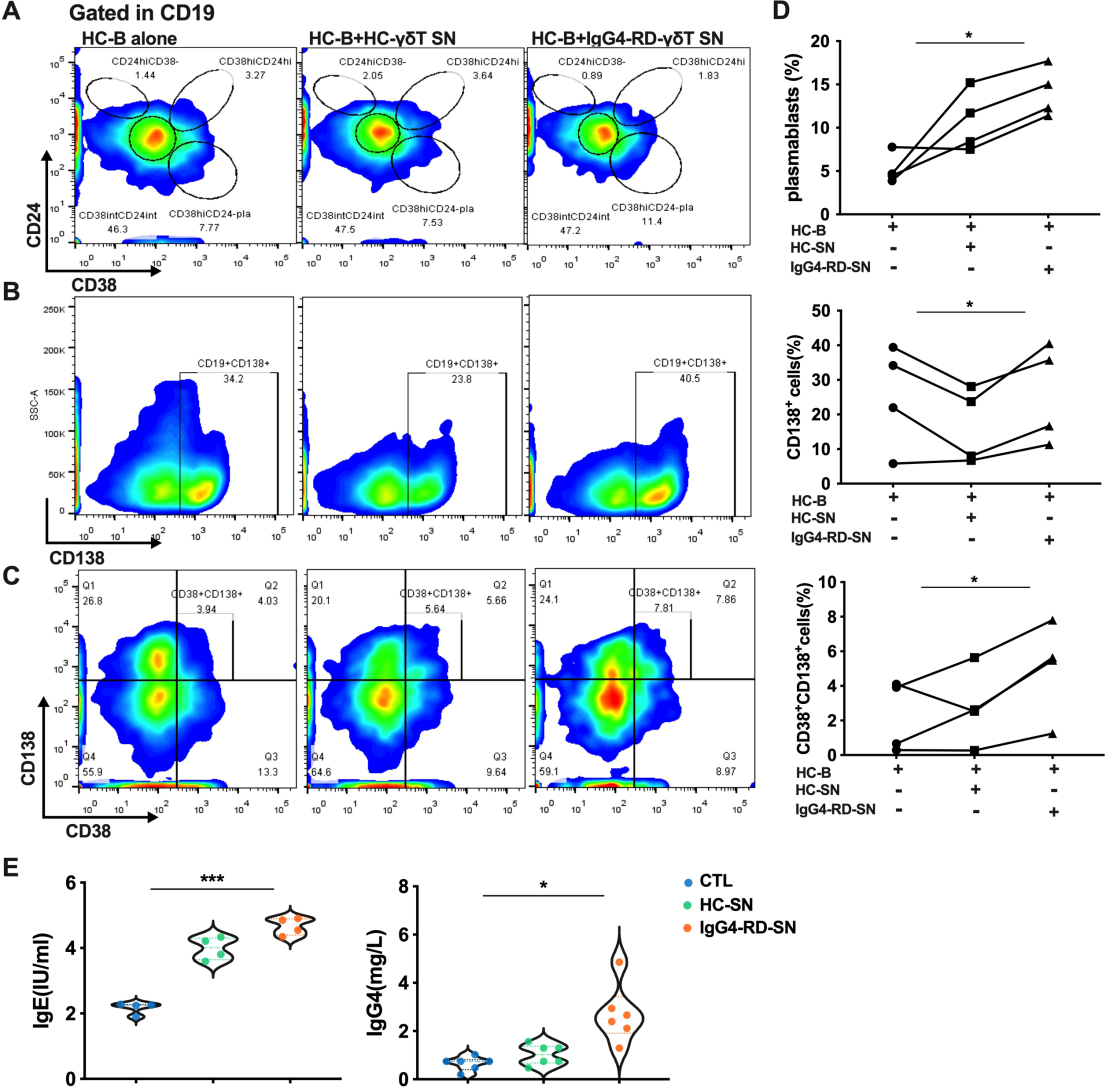
The culture supernatant of Vδ2 T cells from IgG4-RD patients promotes plasmablast differentiation and immunoglobulin expression. **(A)** Flow cytometry analysis showing that the supernatant from Vδ2 T cells (Vδ2 T-SN) of IgG4-RD patients promotes the differentiation of B cells into plasmablasts. **(B)** Flow cytometry analysis indicating that Vδ2 T-SN of IgG4-RD patients increases the proportion of CD138-positive cells among CD19+ B cells. **(C)** Flow cytometry analysis demonstrating that Vδ2 T-SN of IgG4-RD patients promotes the differentiation of B cells into plasma cells. **(D)** ANOVA analysis showing that the supernatant from IgG4-RD Vδ2 T cells (IgG4-RD-SN) induces B cells to differentiate into plasmablasts and plasma cells more effectively than the supernatant from HC Vδ2 T cells (HC-SN) or HC B cell culture alone. **(E)** Violin plots illustrating ELISA results, showing that the IgG4-RD-SN group induces higher levels of antibody secretion (IgE and IgG4) by B cells compared to control groups. SN, culture supernatant; HC-B, B cells from HC; HC-SN, Vδ2 T-SN of HC; IgG4-RD-SN, Vδ2 T-SN of IgG4-RD; CTL, control group; *, P < 0.05; ***, P < 0.001.

We next explored the type 2 immune response in Vδ2 T cells. Supernatants (SN) from IPP-pre-expanded Vδ2 T cells derived from HCs or IgG4-RD patients were added to cultured B cells from HCs. After six days of co-culture, the B cells were analyzed for their differentiation into plasmablasts and for immunoglobulin production. As expected, supernatants from Vδ2 T cells, particularly from patients with active IgG4-RD (aIgG4-RD), strongly induced PB differentiation. This effect was reflected by increased frequencies of CD19+CD24-CD38+, CD19+CD138+, and CD38+CD138+ plasmablasts in the co-cultured B cells (**Figures 6A–D**).

Additionally, immunoglobulin levels were significantly higher in the supernatants of B cells stimulated with Vδ2 T cell-derived SNs from aIgG4-RD patients compared to those stimulated with SNs from HCs. Specifically, IgG4 and IgE levels were significantly elevated (**Figure 6E**). Combined with the high expression of type 2 cytokines in Vδ2 T cells from IgG4-RD patients (**Figure 2B**), these results suggest that Vδ2 T cells in IgG4-RD promote humoral immunity and B-cell activation predominantly through a Th2-like phenotype rather than a Tfh phenotype.

### IL-21–STAT3–Blimp-1 pathway promotes GATA3 expression in Vδ2 T cells and restrained by JAKi/STAT3i

Blimp-1, an IL-21-induced gene, has been reported to promote Th2 differentiation by upregulating GATA3 expression via STAT3 activation (12, 30). Building on this, we investigated whether the IL-21–STAT3–Blimp-1 pathway is activated in Vδ2 T cells from IgG4-RD patients to induce a Th2-like phenotype.

**Figure 7:**
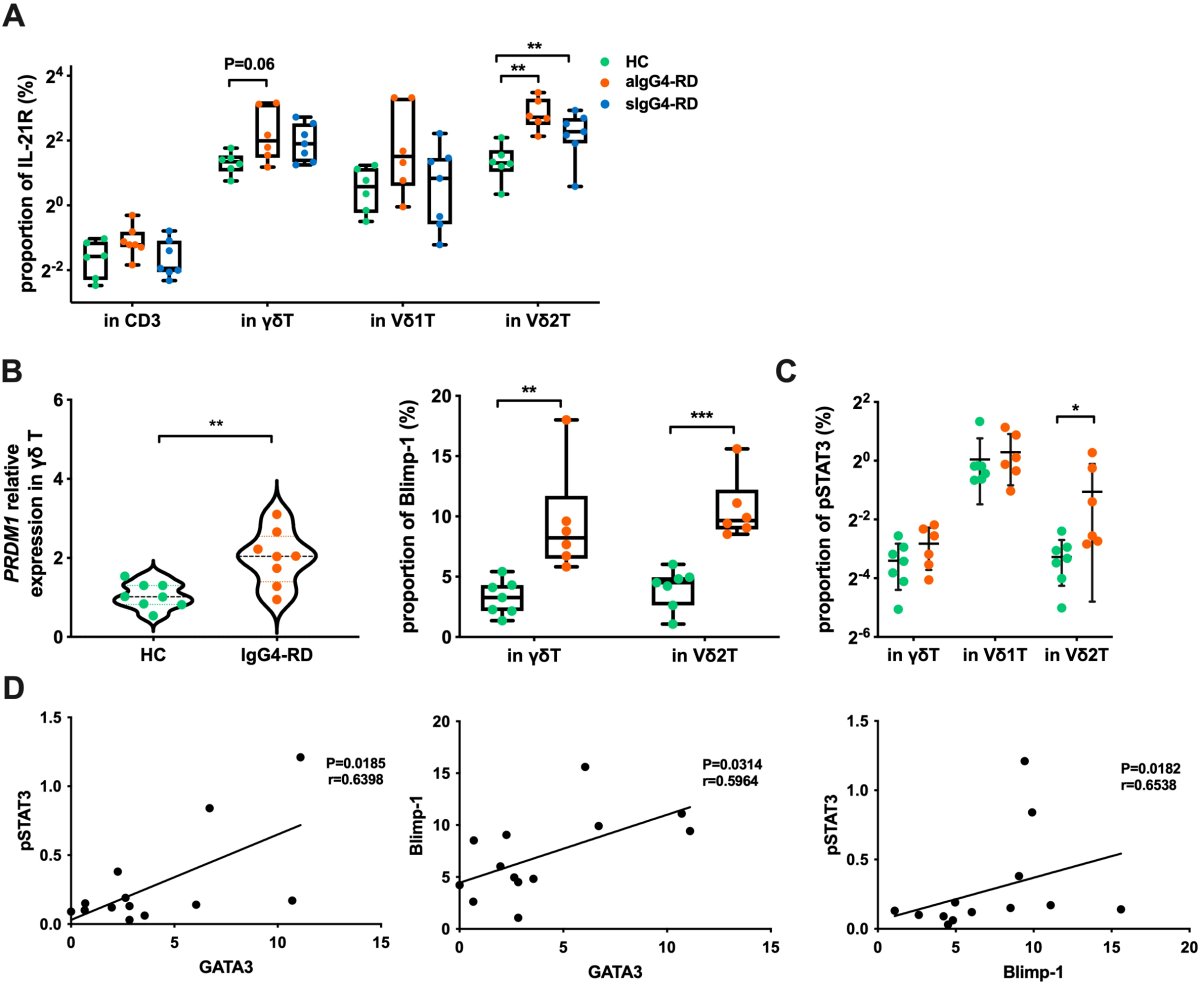
Expression of molecules related to the IL-21 signaling pathway. **(A)** Expression proportion of IL-21R in different cell subsets of healthy controls (HC), active IgG4-RD (aIgG4-RD), and stable IgG4-RD (sIgG4-RD) patients. **(B)** Expression of Blimp-1 at the mRNA level (PRDM1 relative expression) and protein level in HC and IgG4-RD patients. **(C)** Comparison of pSTAT3 expression levels in cell subpopulations detected by flow cytometry. **(D)** Correlation analysis of the expression levels of pSTAT3, Blimp-1, and GATA3 proteins in Vδ2 T cells of IgG4-RD patients. *P < 0.05, **P < 0.01, ***P < 0.001.

When analyzing IL-21 receptor (IL-21R), IL-21-induced genes, and pSTAT3 (pY705), we found that IL-21R, pSTAT3, and Blimp-1 expression levels were significantly higher in Vδ2 T cells from IgG4-RD patients when compared to HCs (p=0.0014, p=0.046, and p=0.0002, respectively), with elevated *PRDM1* expression (encoding Blimp-1) confirmed by RT-PCR (p=0.003) (**Figures 7A–C**). Furthermore, we observed positive correlations between pSTAT3, Blimp-1, and GATA3 in Vδ2 T cells from aIgG4-RD patients (**Figure 7D**). These findings align with the hypothesis that the IL-21R–STAT3–Blimp-1–GATA3 pathway is activated in Vδ2 T cells in aIgG4-RD.

**Figure 8:**
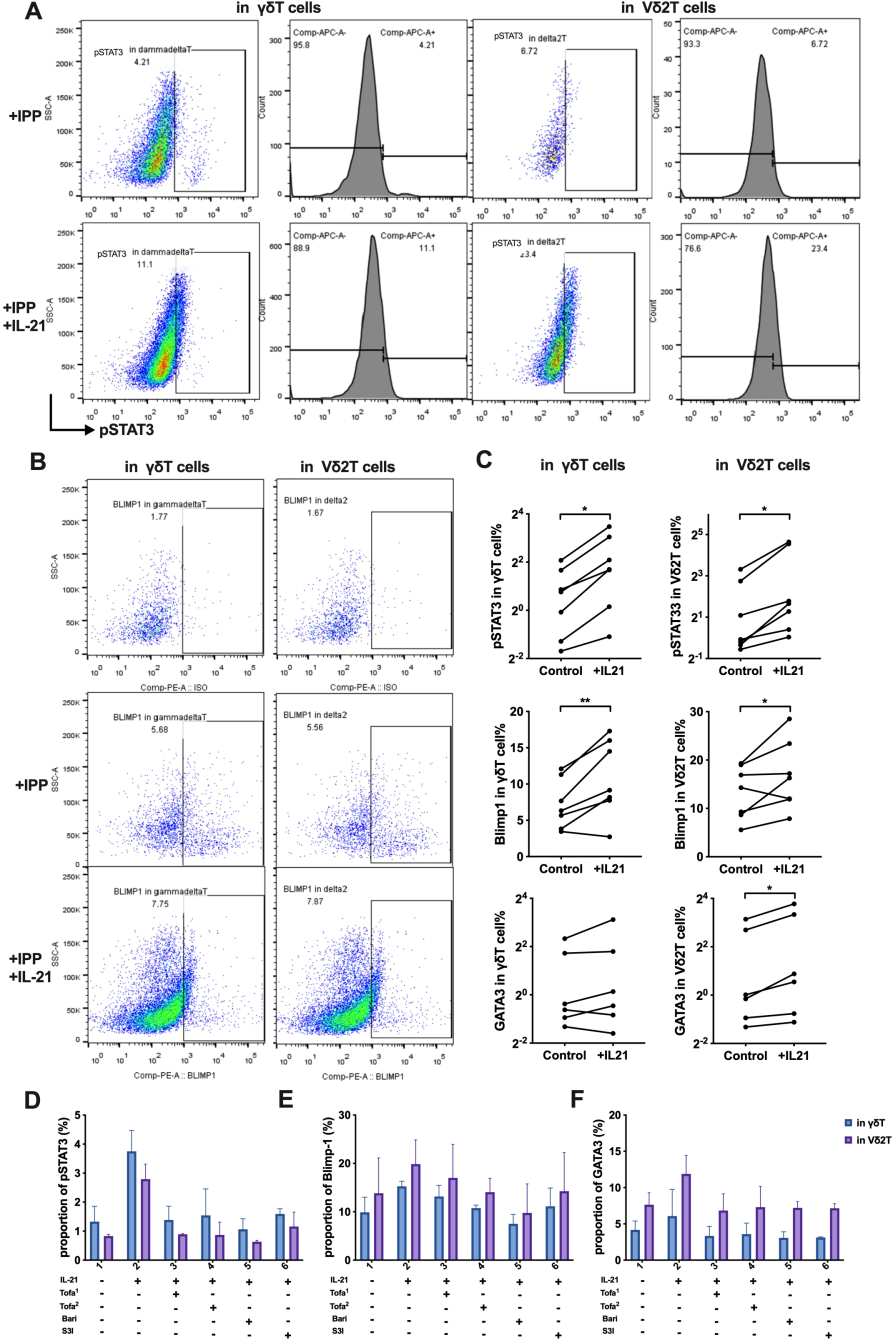
IL-21 induces STAT3—Blimp-1—GATA3 expression, and JAK/STAT3 inhibitors reduce this effect. **(A)** Flow cytometry analysis of the expression proportion of phosphorylated STAT3 (pSTAT3) in γδ T cells and Vδ2 T cells, induced by IL-21. **(B)** Flow cytometry analysis of the expression proportion of Blimp-1 in γδ T cells and Vδ2 T cells, induced by IL-21. **(C)** Comparison of the expression levels of pSTAT3, Blimp-1, and GATA3 proteins between the control group and the IL-21-treated experimental group. Paired t-tests were used to compare the same samples between the control and experimental conditions. **(D-F)** Flow cytometry analysis of the effect of JAK/STAT3 inhibitors on the expression of pSTAT3 **(D)**, Blimp-1**(E)**, GATA3 **(F)**, induced by IL-21 in γδ T cells and Vδ2 T cells. Tofa^1^, Tofacitinib (10 nM); Tofa^2^, Tofacitinib (100 nM); Bari, Baricitinib (300 nM); S3I, STAT3 inhibitor S3I-201 (10 μM). IPP, isopentenyl pyrophosphate; +IL-21, experimental group with IL-21 treatment; Control, control group without IL-21. *P < 0.05, **P < 0.01.

To further evaluate the role of IL-21 in this pathway, we stimulated γδ T cells and IPP-pre-expanded Vδ2 T cells from HCs with or without IL-21. IL-21 stimulation resulted in significantly increased protein levels of pSTAT3, Blimp-1, and GATA3 in Vδ2 T cells (p=0.016, p=0.046, and p=0.031, respectively). In γδ T cells, pSTAT3 and Blimp-1 levels also increased (p=0.026, p=0.009), though GATA3 expression did not (p=0.156) (**Figures 8A–C**).

Building on these results and supported by recent clinical findings (31), we explored whether the IL-21-induced JAK/STAT3 pathway could be pharmacologically targeted. We employed JAK inhibitors, including Baricitinib (JAK1/2 inhibitor) and Tofacitinib (JAK3/1 inhibitor) at two different concentrations. STAT3 was inhibited using S3I-201 (NSC 74859). Treatment with JAK inhibitors suppressed the IL-21-driven increases in pSTAT3, Blimp-1, and GATA3, and this effect was similarly abolished in the presence of the STAT3 inhibitor (**Figures 8D–F**).

In summary, the IL-21–STAT3–Blimp-1 pathway drives GATA3 expression and promotes the Th2-like phenotype of Vδ2 T cells in IgG4-RD. Targeting this pathway with JAK or STAT3 inhibitors could provide a therapeutic strategy to attenuate the pathogenic properties of Vδ2 T cells in this disease.

## Discussion

This study demonstrated a reduced proportion of circulating Vδ2 T cells but increased infiltration of these cells in affected organs of patients with IgG4-RD. Vδ2 T cells from IgG4-RD patients exhibited elevated expression of type 2 cytokines and the chemotactic receptor CCR7. Consistently, we observed co-localization of Vδ2 T cells with CCR7/CCL21 and B cells in affected tissues, suggesting that peripheral Vδ2 T cells migrate into involved tissues via the CCR7/CCL21 axis and interact with B cells. Using a co-culture model, we found that Vδ2 T cells from IgG4-RD patients were more efficient at promoting B cell differentiation and immunoglobulin production compared to those from healthy controls. These effects were predominantly mediated by type 2 cytokines. Moreover, our data revealed a previously unappreciated role of the IL-21–STAT3–Blimp-1 pathway as an initiator of the Th2 response in Vδ2 T cells, which could be inhibited by targeting the JAK/STAT3 signaling pathway.

A reduction in the Vδ2 T cell population has also been reported in other autoimmune conditions, such as systemic lupus erythematosus (SLE) (32) and rheumatoid arthritis (RA) (27). While Vδ2 T cells typically produce pro-inflammatory cytokines, such as interferon (IFN)-γ and tumor necrosis factor (TNF)-α, they can deviate from a Th1-like phenotype and adopt alternative effector profiles, including Th2, Th17, Tfh, and Treg, depending on the pathophysiological context (33, 34). For instance, Vδ2 T cells have been shown to support plasmablast differentiation through a Tfh-like phenotype (35). In this study, we identified a Th2-like Vδ2 T cell population in IgG4-RD, which aligns with the type 2 immune responses commonly associated with this disease. IgG4-RD shares several features with allergic diseases, such as type 2 immune responses, IgG4 and IgE hypersecretion, and blood/tissue eosinophilia (3). However, this is the first study to demonstrate the involvement of Vδ2 T cells in the pathogenesis of IgG4-RD through a Th2-like phenotype.

Interestingly, Vδ2 T cells in IgG4-RD did not exhibit a robust Tfh-like phenotype, such as CD40L, ICOS, BCL-6, CXCR5, and IL-21. Instead, these cells supported B cell differentiation and antibody production *in vitro* primarily through type 2 cytokine secretion. However, we acknowledge that the absence of significant differences in IL-21 expression does not exclude its potential functional role in B cell differentiation. IL-21 is a well-documented cytokine in IgG4-RD, particularly in driving B cell differentiation (13). Thus, while our findings suggest that Vδ2 T cells predominantly adopt a Th2-like phenotype, they do not exclude the possibility of Tfh-like functions or IL-21 contributing under specific conditions. Future studies using blocking antibodies targeting IL-4, IL-21, or their receptors will be essential to validate the specific contributions of these cytokines to the Th2-like phenotype and B cell activation. In addition to type 2 cytokine production, we found increased expression of collagen-related genes in γδ T cells from IgG4-RD patients, suggesting a profibrotic phenotype likely mediated indirectly through the secretion of type 2 cytokines and TGF-β. However, similar to B cells (22), which are known to directly contribute to fibrosis in IgG4-RD, Vδ2 T cells may also have a direct role. Future studies should focus on utilizing co-culture systems to evaluate the direct effect of Vδ2 T cells on fibroblast activation and collagen synthesis.

Increasing evidence suggests that Vδ2 T cells represent a heterogeneous population capable of generating cytokine-skewed immune responses, reflecting the influence of the microenvironment during differentiation (9). Blimp-1, a key IL-21 target gene, has been reported to be upregulated by STAT3 activation and to promote Th2 cell development by inducing GATA3 expression (30). Using γδ T cells from healthy individuals, we established and validated the IL-21/STAT3/Blimp-1/GATA3 pathway, demonstrating that stimulation with IL-21 induced a Th2-like phenotype. Targeting the JAK/STAT3 pathway effectively suppressed this effect, consistent with previous studies reporting that JAK inhibitors are potentially effective treatments for IgG4-RD and allergic diseases (31, 36). Notably, Baricitinib, a JAK1/2 inhibitor, exhibited stronger suppression of the IL-21-driven Th2-like phenotype compared to Tofacitinib (JAK3/1 inhibitor), likely due to its additional inhibition of JAK2 to block IL-21–STAT3 signaling more comprehensively.

We acknowledge that the microenvironment in IgG4-RD may not only impact Vδ2 T cells but could also reshape other T cell subsets, such as Th2, Tfh, and Tph cells. Therefore, the Th2-like phenotype observed in Vδ2 T cells may not purely reflect an intrinsic differentiation program but could rather represent a broader phenomenon of T cell plasticity. The functional overlap between Vδ2 T cells and pre-existing Th2 subsets points to a potential bystander effect, where a microenvironment dominated by these abundant subsets indirectly shapes Vδ2 T cell function. However, our study specifically highlights the ability of Vδ2 T cells to directly respond to IL-21 and adopt a Th2-like phenotype, as shown *in vitro*. Future studies, such as single-cell transcriptomic analyses of affected tissues, are needed to clarify whether their Th2-like phenotype reflects intrinsic differentiation or a response to IL-21.

In addition to the IL-21-driven microenvironment, Vδ2 T cells respond to phosphoantigens (pAgs) produced by cellular pathogens, and immune challenges can lead to a decrease in Vδ2 T cells and a reversal of the Vδ2/Vδ1 ratio (8, 37). Our previous work identified a higher frequency of pathogen-associated TCR sequences in T cells from IgG4-RD patients (38). These findings suggest a potential association between Vδ2 T cells and pathogen exposure in IgG4-RD, which warrants further investigation.

γδ T cells have been shown to migrate to local tissues in autoimmune diseases, contributing to inflammation and tissue injury through specific chemokine receptors (27, 32, 39, 40). In this study, we observed significantly upregulation of CCR7 on Vδ2 T cells from IgG4-RD patients, consistent with previous findings that IL-21 sustains CCR7 expression on the cell surface (12, 41). Our data suggest that peripheral Vδ2 T cells infiltrate IgG4-RD lesions, at least in part through the CCR7/CCL21 axis, and may interact with infiltrating B cells. However, these findings likely reflect a broader and more complex chemokine network. For instance, CCL19 may also mediate Vδ2 T cell migration, and the broad expression of CCR7 on other T cell subsets suggests that additional cells could respond to CCL19/CCL21 signaling. Additionally, B cells in IgG4-RD may share overlapping chemokine receptors with Vδ2 T cells, indicating that other pathways beyond the CCR7/CCL21 axis might contribute to their interactions in affected tissues.

In conclusion, this study highlights the role of γδ T cells in the pathogenesis of IgG4-RD. Vδ2 T cells in IgG4-RD exhibited a Th2-like phenotype and demonstrated an enhanced ability to induce plasmablast differentiation and antibody production. Targeting JAK or STAT3 signaling effectively inhibited the Th2-like phenotype in Vδ2 T cells, providing potential therapeutic implications for IgG4-RD. These findings not only underscore the importance of Vδ2 T cells in IgG4-RD but also pave the way for future investigations into novel treatment strategies targeting γδ T cells and their associated pathways.

## Data Availability

The original contributions presented in the study are included in the article/**Supplementary Material**. Further inquiries can be directed to the corresponding authors.
