# Supplementary material for: Polarization of Vδ2 T cells to a Th2-like phenotype promotes plasmablast differentiation and possesses pro-fibrotic properties in IgG4-related disease
Source: Front Immunol. 2025 Mar 27;16:1550405. doi: 10.3389/fimmu.2025.1550405 (PMC11983612; doi:10.3389/fimmu.2025.1550405)
Supplement: Supplementary file 1 [file DataSheet1.docx]

**Supplementary figures:**

**
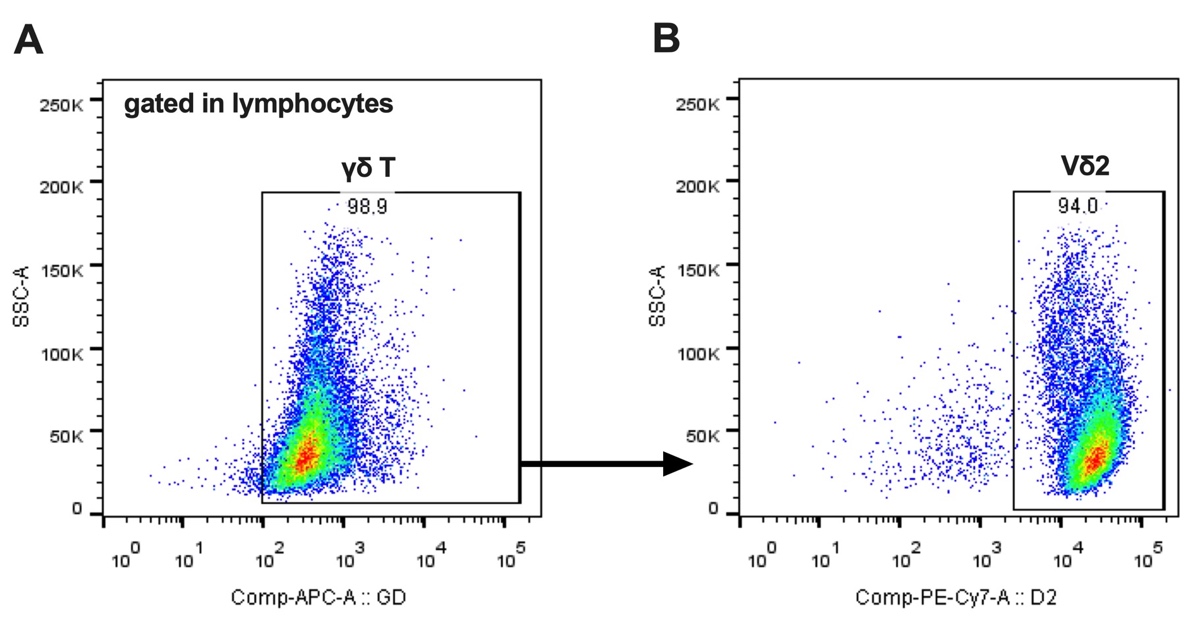
Figure S1:** (A) The purity of isolated γδ T cells. (B) The purity of Vδ2 T cells after pre-expansion.


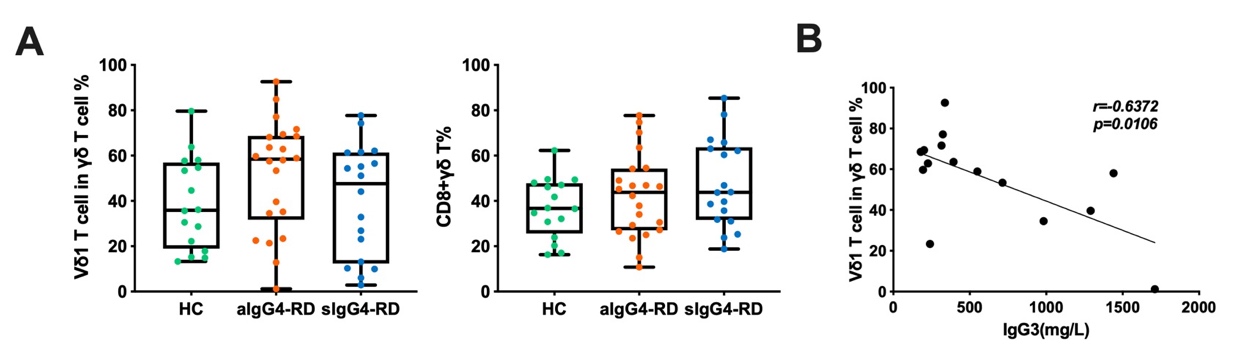


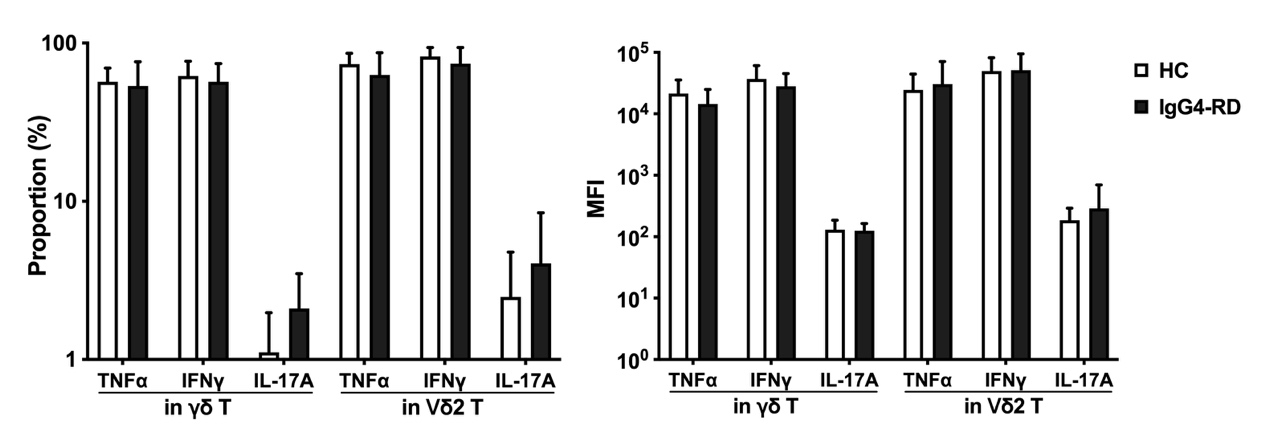
**Figure S2:** (A) Comparison of the percentages of Vδ1 T cells and CD8+γδ T cells in patients with active IgG4-RD (aIgG4-RD), stable IgG4-RD (sIgG4-RD), and healthy controls (HC). (B) Negative correlation between the proportion of Vδ1 T cells and serum IgG3 level.

**Figure S3:** The percentage and MFI of Th1 cytokines including TNF-α, IFN-γ, and IL-17A expressed in γδ T cells and Vδ2 T cells from HC and IgG4-RD patients.


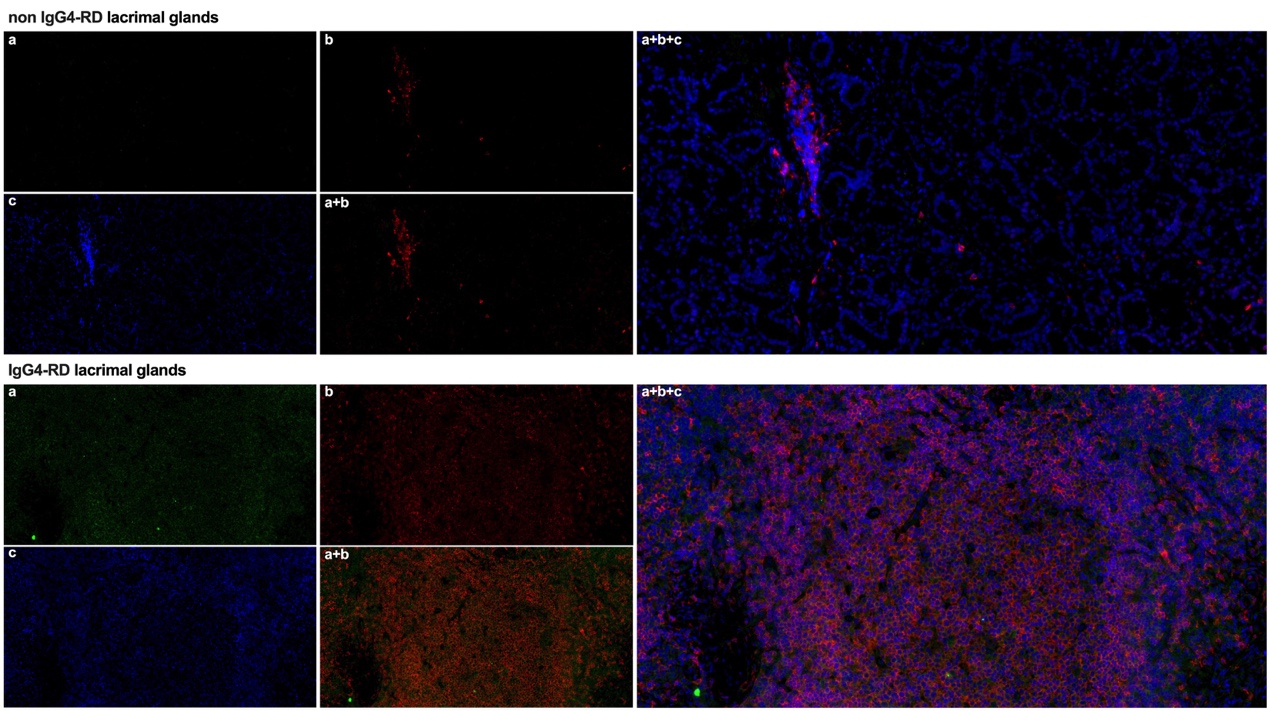
**Figure S4:** Immunofluorescence results showing that lacrimal glands of IgG4-RD patients have more Vδ2 T cells infiltrating, and these Vδ2 T cells colocalize with B cells compared to the lacrimal gland tissues of patients with lacrimal gland prolapse. a, Vδ2 T (green); b, CD20 (red); c, DAPI counterstaining for cell nuclei (blue); a + b, Merged image of Vδ2 T and CD20; a + b + c, Merged image of Vδ2 T, CD20, and DAPI; Magnification: 20×.

**
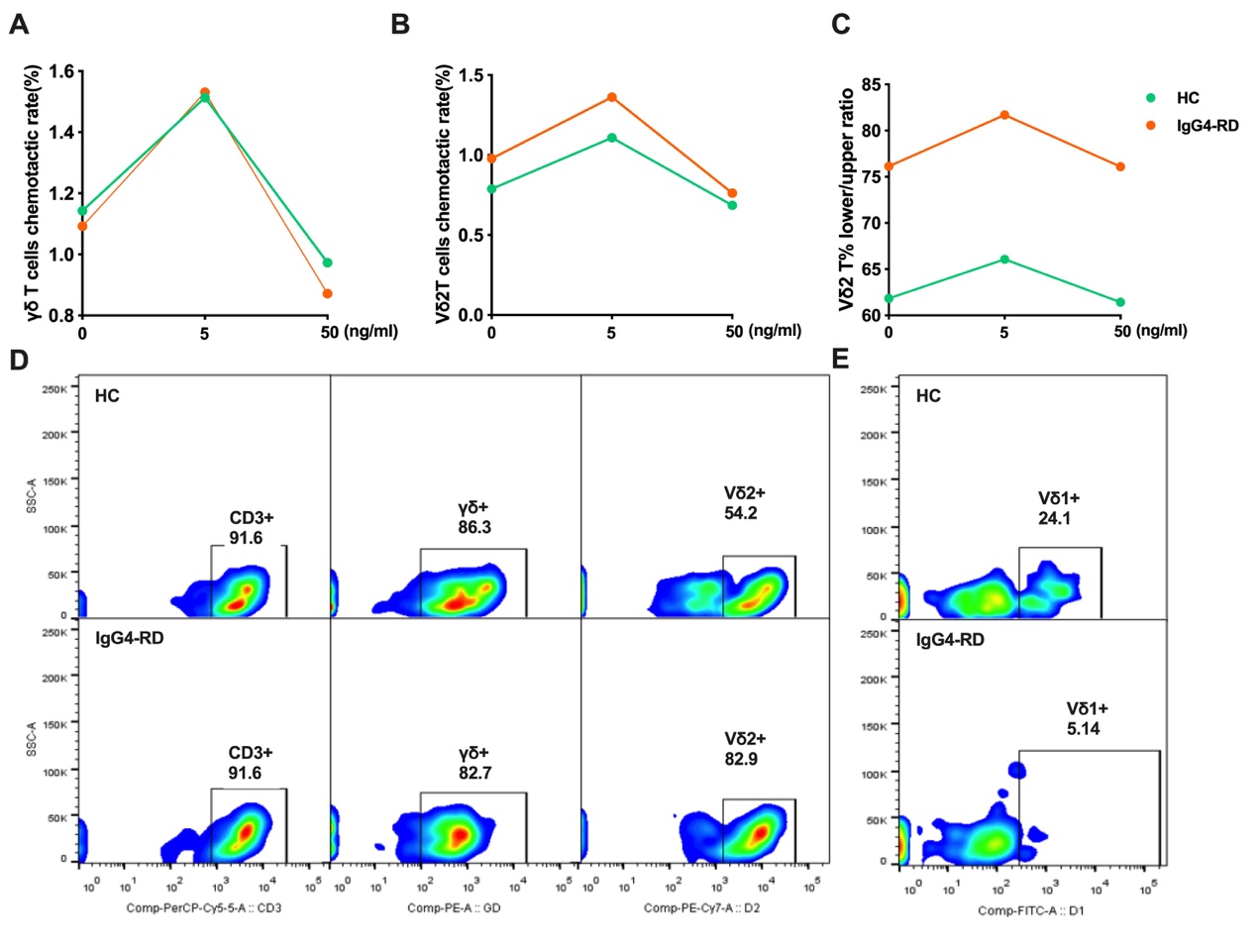
Figure S5:** Transwell migration assay. (A) The chemotaxis rate of γδ T cells derived from IgG4-RD patients and HCs compared under different concentrations of CCL21. (B) The chemotaxis rate of Vδ2 T cells derived from IgG4-RD patients and HCs compared under different concentrations of CCL21. (C) The ratio of the percentage of Vδ2 T cells in the lower chamber to that in the upper chamber. (D) Flow cytometry showing the percentage of Vδ2 T cells in the lower chamber. (E) Flow cytometry showing the percentage of Vδ1 T cells in the lower chamber.


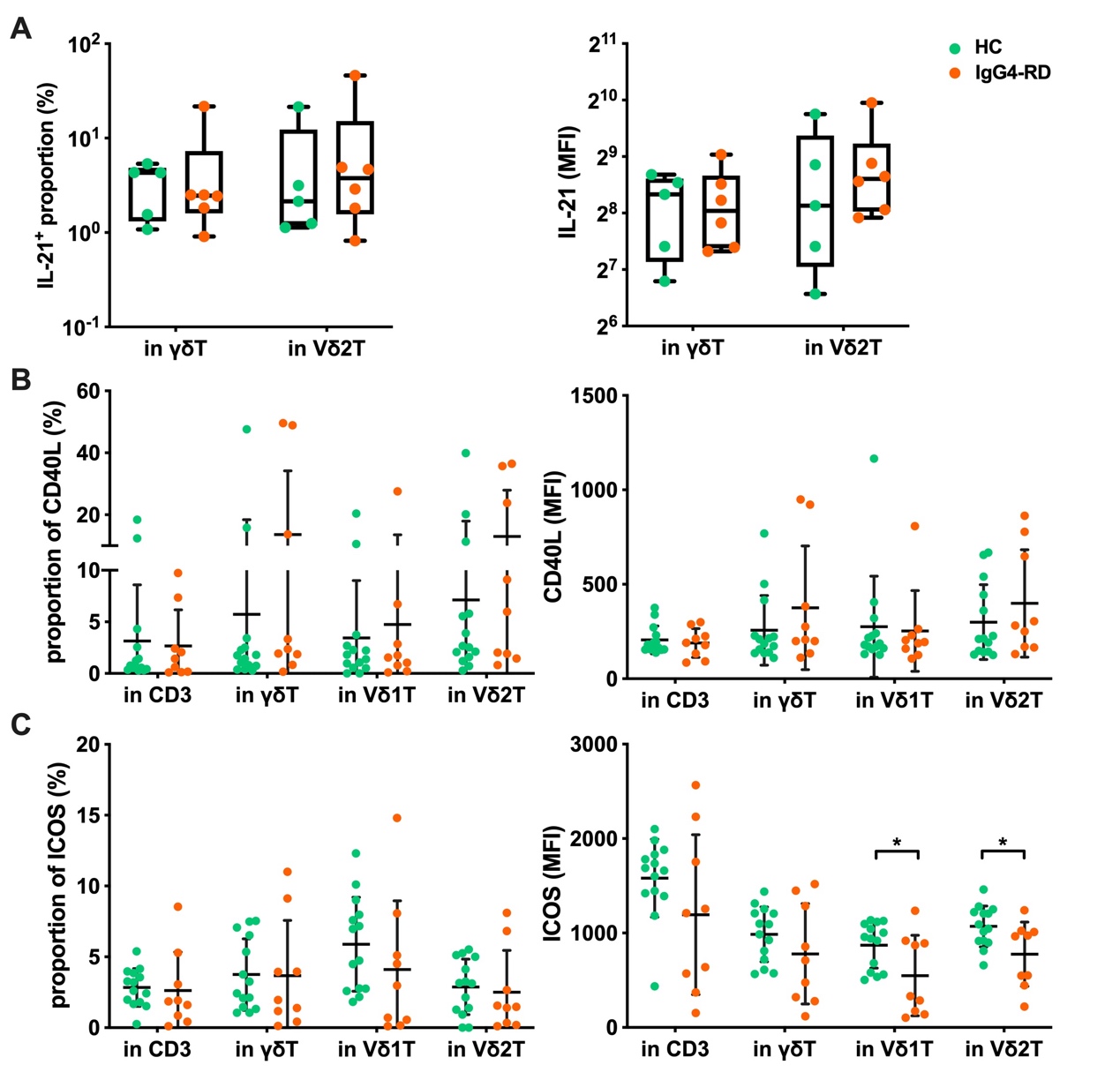


**Figure S6:** Expression of CD40L, ICOS, and IL-21 in different cell subsets. (A) The percentage and MFI of IL-21 in γδ T cells and Vδ2 T cells from HC and IgG4-RD patients. (B) The percentage and MFI of CD40L in CD3+ T cells, γδ T cells, and their subsets from HC and IgG4-RD patients. (C) The percentage and MFI of ICOS in CD3+ T cells, γδ T cells, and their subsets from HC and IgG4-RD patients. ICOS, Inducible costimulatory; *, P < 0.05.

**Supplementary tables:**

Table S1. Clinical characteristics of 40 patients with IgG4-RD

| **Characteristic** | **N = 40***^1^* |
| --- | --- |
| Male gender | 22 (55%) |
| Age (years) | 63 (53, 70) |
| Allergy | 22 (55%) |
| **Number of organs affected** |  |
| 1 | 10 (26%) |
| 2 | 6 (16%) |
| 3 | 10 (26%) |
| 4 | 8 (21%) |
| 5 | 2 (5.3%) |
| 6 | 2 (5.3%) |
| **Laboratory parameters** |  |
| EOS (10^9/L) | 0.21 (0.12, 0.41) |
| IgG (g/L) | 22 (17, 30) |
| IgG4 (mg/L) | 12,900 (4,458, 24,575) |
| T-IgE (KU/L) | 442 (152, 758) |
| *^1^*n (%); Median (IQR); EOS, eosinophils | |

Table S2. List of Flow Cytometry Antibodies Used in the Study

| **Category** | **Marker** | **Fluorochrome** | **Antibody Source** |
| --- | --- | --- | --- |
| **T Cells and Subsets** | CD3 | FITC, PerCP/Cy5.5 | BD Biosciences |
|  | TCR γδ | PE, APC | BD Biosciences |
|  | TCR Vδ1 | FITC | Invitrogen |
|  | TCR Vδ2 | PeCy7, FITC | BD Biosciences |
| **Chemokine Receptors** | CCR5 | PE | BD Biosciences |
|  | CCR6 | FITC | BD Biosciences |
|  | CCR7 | APC | BD Biosciences |
|  | CCR8 | PE | BD Biosciences |
|  | CXCR3 | APC | BD Biosciences |
|  | CXCR5 | PerCP/Cy5.5 | BD Biosciences |
| **Intracellular Cytokines** | TNF-α | APC | BD Biosciences |
|  | IFN-γ | APC | BD Biosciences |
|  | IL-17A | PE | BD Biosciences |
|  | IL-4 | PE | BD Biosciences |
|  | IL-5 | PE | BD Biosciences |
|  | IL-6 | APC | BD Biosciences |
|  | IL-9 | PE | BD Biosciences |
|  | IL-10 | APC | BD Biosciences |
|  | IL-13 | PE | BD Biosciences |
|  | IL-21 | APC | BD Biosciences |
|  | IL-22 | FITC | BD Biosciences |
|  | TGF-β | APC | BD Biosciences |
| **Phosphorylated Proteins** | Blimp-1 | PE | BD Biosciences |
|  | GATA3 | PE | BD Biosciences |
|  | Phospho-STAT3 | PE, APC | BD Biosciences, BioLegend |
| **B Cells** | CD19 | PeCy7 | BD Biosciences |
|  | CD24 | FITC | BD Biosciences |
|  | CD38 | APC | BD Biosciences |
|  | CD138 | PE | BD Biosciences |
|  | IgD | PE | BD Biosciences |
|  | CD27 | PerCP/Cy5.5 | BD Biosciences |
| **Other Surface Molecules** | CD40L | PE | BD Biosciences |
|  | ICOS | APC | BD Biosciences |
|  | IL-21R | PE | BD Biosciences |
| **Controls** | Isotype Ctrl | Various | BD Biosciences, BioLegend, Invitrogen |

Table S3. List of primers used for RT-PCR

| Target | Forward Primer (5’-3’) | Reverse Primer (5’-3’) |
| --- | --- | --- |
| TBX21 | GCCCACGATGAAACCTGAGA | GCTCCTTCATGCCCAAGACT |
| GATA3 | GGCGAACTCTGCCTGTCATT | ACGACTCTGCAATTCTGCGA |
| RORC | AAGAAGACCCACACCTCACA | TGCACCCCTCACAGGTGATA |
| FOXP3 | GAAGGACAGGTCAGTGGACAG | CCATTTGCCAGCAGTGGGTA |
| BCL6 | AACCTGAAAACCCACACTCG | TTCGCATTTGTAGGGCTTCT |
| PRDM1 | GTGTCAGAACGGGATGAACA | GCTCGGTTGCTTTAGACTGC |
| β-actin | CCTGGGCATGGAGTCCTGTGG | CTGTGTTGGCGTACAGGTCTT |
| hCOL1A1 | CACACGTCTCGGTCATGGTA | CGGCTCCTGCTCCTCTTAG |
| hCOL1A2 | AGCAGGTCCTTGGAAACCTT | GAAAAGGAGTTGGACTTGGC |
| hCOL3A1 | ATATTTGGCATGGTTCTGGC | TGGCTACTTCTCGCTCTGCT |
| hACTA2 | GATGGCCACTGCCGCATCCT | ACAGGGTCTCTGGGCAGCGG |
| hGAPDH | GGTGAAGGTCGGAGTCAACGGA | GAGGGATCTCGCTCCTGGAAGA |
