# Supplementary material for: Polarization of Vδ2 T cells to a Th2-like phenotype promotes plasmablast differentiation and possesses pro-fibrotic properties in IgG4-related disease
Source: Front Immunol. 2025 Mar 27;16:1550405. doi: 10.3389/fimmu.2025.1550405 (PMC11983612; doi:10.3389/fimmu.2025.1550405)
Supplement: Supplementary file 2 [file Table1.docx]

**Supplementary tables:**

Table S1. Clinical characteristics of 40 patients with IgG4-RD

| **Characteristic** | **N = 40***^1^* |
| --- | --- |
| Male gender | 22 (55%) |
| Age (years) | 63 (53, 70) |
| Allergy | 22 (55%) |
| **Number of organs affected** |  |
| 1 | 10 (26%) |
| 2 | 6 (16%) |
| 3 | 10 (26%) |
| 4 | 8 (21%) |
| 5 | 2 (5.3%) |
| 6 | 2 (5.3%) |
| **Laboratory parameters** |  |
| EOS (10^9/L) | 0.21 (0.12, 0.41) |
| IgG (g/L) | 22 (17, 30) |
| IgG4 (mg/L) | 12,900 (4,458, 24,575) |
| T-IgE (KU/L) | 442 (152, 758) |
| *^1^*n (%); Median (IQR); EOS, eosinophils | |

Table S2. List of Flow Cytometry Antibodies Used in the Study

| **Category** | **Marker** | **Fluorochrome** | **Antibody Source** |
| --- | --- | --- | --- |
| **T Cells and Subsets** | CD3 | FITC, PerCP/Cy5.5 | BD Biosciences |
|  | TCR γδ | PE, APC | BD Biosciences |
|  | TCR Vδ1 | FITC | Invitrogen |
|  | TCR Vδ2 | PeCy7, FITC | BD Biosciences |
| **Chemokine Receptors** | CCR5 | PE | BD Biosciences |
|  | CCR6 | FITC | BD Biosciences |
|  | CCR7 | APC | BD Biosciences |
|  | CCR8 | PE | BD Biosciences |
|  | CXCR3 | APC | BD Biosciences |
|  | CXCR5 | PerCP/Cy5.5 | BD Biosciences |
| **Intracellular Cytokines** | TNF-α | APC | BD Biosciences |
|  | IFN-γ | APC | BD Biosciences |
|  | IL-17A | PE | BD Biosciences |
|  | IL-4 | PE | BD Biosciences |
|  | IL-5 | PE | BD Biosciences |
|  | IL-6 | APC | BD Biosciences |
|  | IL-9 | PE | BD Biosciences |
|  | IL-10 | APC | BD Biosciences |
|  | IL-13 | PE | BD Biosciences |
|  | IL-21 | APC | BD Biosciences |
|  | IL-22 | FITC | BD Biosciences |
|  | TGF-β | APC | BD Biosciences |
| **Phosphorylated Proteins** | Blimp-1 | PE | BD Biosciences |
|  | GATA3 | PE | BD Biosciences |
|  | Phospho-STAT3 | PE, APC | BD Biosciences, BioLegend |
| **B Cells** | CD19 | PeCy7 | BD Biosciences |
|  | CD24 | FITC | BD Biosciences |
|  | CD38 | APC | BD Biosciences |
|  | CD138 | PE | BD Biosciences |
|  | IgD | PE | BD Biosciences |
|  | CD27 | PerCP/Cy5.5 | BD Biosciences |
| **Other Surface Molecules** | CD40L | PE | BD Biosciences |
|  | ICOS | APC | BD Biosciences |
|  | IL-21R | PE | BD Biosciences |
| **Controls** | Isotype Ctrl | Various | BD Biosciences, BioLegend, Invitrogen |

Table S3. List of primers used for RT-PCR

| Target | Forward Primer (5’-3’) | Reverse Primer (5’-3’) |
| --- | --- | --- |
| TBX21 | GCCCACGATGAAACCTGAGA | GCTCCTTCATGCCCAAGACT |
| GATA3 | GGCGAACTCTGCCTGTCATT | ACGACTCTGCAATTCTGCGA |
| RORC | AAGAAGACCCACACCTCACA | TGCACCCCTCACAGGTGATA |
| FOXP3 | GAAGGACAGGTCAGTGGACAG | CCATTTGCCAGCAGTGGGTA |
| BCL6 | AACCTGAAAACCCACACTCG | TTCGCATTTGTAGGGCTTCT |
| PRDM1 | GTGTCAGAACGGGATGAACA | GCTCGGTTGCTTTAGACTGC |
| β-actin | CCTGGGCATGGAGTCCTGTGG | CTGTGTTGGCGTACAGGTCTT |
| hCOL1A1 | CACACGTCTCGGTCATGGTA | CGGCTCCTGCTCCTCTTAG |
| hCOL1A2 | AGCAGGTCCTTGGAAACCTT | GAAAAGGAGTTGGACTTGGC |
| hCOL3A1 | ATATTTGGCATGGTTCTGGC | TGGCTACTTCTCGCTCTGCT |
| hACTA2 | GATGGCCACTGCCGCATCCT | ACAGGGTCTCTGGGCAGCGG |
| hGAPDH | GGTGAAGGTCGGAGTCAACGGA | GAGGGATCTCGCTCCTGGAAGA |
